# ATTRACT-EM: A New Method for the Computational Assembly of Large Molecular Machines Using Cryo-EM Maps

**DOI:** 10.1371/journal.pone.0049733

**Published:** 2012-12-14

**Authors:** Sjoerd J. de Vries, Martin Zacharias

**Affiliations:** Physik-Department T38, Technische Universität München, Garching, Germany; Indian Institute of Science, India

## Abstract

Many of the most important functions in the cell are carried out by proteins organized in large molecular machines. Cryo-electron microscopy (cryo-EM) is increasingly being used to obtain low resolution density maps of these large assemblies. A new method, ATTRACT-EM, for the computational assembly of molecular assemblies from their components has been developed. Based on concepts from the protein-protein docking field, it utilizes cryo-EM density maps to assemble molecular subunits at near atomic detail, starting from millions of initial subunit configurations. The search efficiency was further enhanced by recombining partial solutions, the inclusion of symmetry information, and refinement using a molecular force field. The approach was tested on the GroES-GroEL system, using an experimental cryo-EM map at 23.5 Å resolution, and on several smaller complexes. Inclusion of experimental information on the symmetry of the systems and the application of a new gradient vector matching algorithm allowed the efficient identification of docked assemblies in close agreement with experiment. Application to the GroES-GroEL complex resulted in a top ranked model with a deviation of 4.6 Å (and a 2.8 Å model within the top 10) from the GroES-GroEL crystal structure, a significant improvement over existing methods.

## Introduction

Proteins are the clockwork of the complex molecular machinery that underlies human life [1]. Numerous diseases, including cancer, Alzheimer and AIDS, can be directly attributed to mechanisms operating at the protein level. Many of the most important functions in the cell are carried out by proteins organized in molecular machines: large, dynamic, macromolecular assemblies such as the ribosome, the proteasome, the spliceosome and the nuclear pore complex [2]–[5]. A mechanistic, atomic-resolution understanding of molecular machines is needed for rational drug design against the diseases associated with their mechanisms. Unfortunately, atomic-resolution techniques such as X-ray crystallography and Nuclear Magnetic Resonance (NMR) are often difficult to apply to large and dynamic macromolecular assemblies, implicating that other techniques are necessary.

Over the last decades, cryo-electron microscopy (cryo-EM) has emerged as an important technique in the study of these molecular machines [6]–[8]. Like crystallography, cryo-EM ultimately produces a three-dimensional map where the value of each voxel is proportional to the electron density. Unfortunately, cryo-EM maps typically have a much lower resolution than crystallographic maps. Still, insight at the atomic level can be obtained if the molecular machine can be assembled computationally from pre-existing atomic structures using the cryo-EM map [9]–[11]. In general, two approaches are possible. When a density map of sufficiently high resolution and a good initial estimate of the assembly structure are available, flexible fitting can be attempted [6]–[16]. Otherwise, however, one has to resort to *de novo* assembly of the individual components. Many different algorithms have been developed for sequential, rigid fitting of single components into cryo-EM maps [6], [17]–[28].

Many rigid fitting methods use simplified, feature-based representations of the protein components that are fitted into the density map. Typically, clustering and spatial feature detection reduces both the protein and the cryo-EM map to a number of centroids, Gaussians or other feature points (feature-to-feature fitting) [6], [19], [29]–[31]. Alternatively, in the COLORES method [20], the density map is kept but the protein is converted to a grid representation, which is overlaid onto the density map grid (grid-to-grid fitting). The simplified protein representations used in rigid fitting methods are in contrast to flexible fitting methods, which typically preserve full atomic representation of the protein (atom-to-grid fitting) [7]–[15]. However, the atom-to-grid fitting approach is also taken by some rigid fitting methods [21].

At lower map resolutions, the sequential fitting of components has the disadvantage that a component can simply drift to the center of a large electron density map [20]. To overcome this problem, COLORES has a contour-matching (Laplacian) mode, which replaces the electron density map with a map that contains the magnitudes of the electron density curvature. Still, there are limits to sequential fitting [30], [31], and contour-matching can overcome them only to a certain extent [32].

More recently, several methods have appeared that fit multiple (rigid) components simultaneously into an electron density map, in particular MultiFit [30], GMFit [29], and IQP [31]. These methods are based on feature detection, and perform very well on assemblies with few (2–7) components, using simulated electron density data. In addition, in the recent version of Situs, a multi-component steepest ascent method has been developed, aimed at the refinement of previously placed models in a visual environment [33].

However, the assembly of molecular machines into cryo-EM maps is a very difficult problem, and the amount of progress so far has been very limited. It is a useful computational exercise to take the components of a crystallized complex and re-assemble them (bound docking), using a cryo-EM density map simulated from the crystallized complex. However, in a real-life situation, neither simulated cryo-EM density nor the bound coordinates of the components would be available. Also, the molecular machine would be large, because cryo-EM currently has a lower size limit of hundreds of KDa. Unfortunately, all three simultaneous assembly methods have only been successful for simulated cryo-EM data, and only MultiFit has been applied to a system without using the bound coordinates.

The bacterial chaperone GroES-GroEL is an excellent model system for large molecular machines. It consists of 21 components arranged in three symmetric homoheptameric rings: GroES, the GroEL cis ring and the GroEL trans ring. The assembly has been crystallized as a whole (PDB code 1AON [34]), providing a reference state to which assembled models can be compared. In addition, several cryo-electron maps for GroES-GroEL have been obtained [35]–[38]; of these, the 23.5 Å map by Ranson et al. [35] (EM databank [39], [40] code 1046) has been used in several studies as a test case for rigid fitting [23], [24], [29], [31]. Unfortunately, the components of GroES and GroEL have not been crystallized in monomeric forms, making it still necessary to use the bound coordinates from 1AON.

In contrast with smaller assemblies, GroES-GroEL has proven to be a very challenging case, and previous attempts to assemble it into the EMD-1046 map have had only limited success. Assembling just the GroEL cis-ring, the IQP method [31] achieved an accuracy of 8.6 Å root-mean square deviation (RMSD), whereas GMFit [29] was used in an attempt to assemble the full GroES-GroEL, achieving an accuracy of 14.7 Å RMSD, with the GroES ring completely flipped. This suggests that when it comes to assembling large molecular machines using experimental cryo-EM density maps (rather than small assemblies with simulated density), there is considerable room for improvement, even for computational exercises like GroES-GroEL.

Protein-protein docking is the computational discipline that tries to assemble molecular machines from their protein subunits, with or without experimental data. Like cryo-EM fitting methods, docking methods can use either simplified [41]–[45] or atom-based representations [46]–[52], [53]. A simplified representation allows a global, exhaustive search, while atom-based docking methods rely on local, heuristic searches using either energy minimization or a Monte Carlo approach. However, atom-based docking methods are inherently flexible, in the sense that additional energy terms (including, but certainly not limited to, actual atomic flexibility) can easily be added to the docking framework.

In recent years, docking methods have made considerable progress in the blind prediction of protein complexes [54], [55]. In fact, since docking scores often have a physical basis, there is an active interest in their application to other biophysical problems, such as protein design and the prediction of binding affinities [56]–[58]. For cryo-EM fitting, there is a need for methods that efficiently explore both conformational and configurational space [59], and large and flexible complexes remain a formidable challenge for docking programs [60]. Therefore, integration of cryo-EM fitting and protein-protein docking methodology might be beneficial, especially for very large and dynamic molecular machines where either method alone fails.

Here we present ATTRACT-EM, a new computational method for the simultaneous assembly of multiple components into cryo-EM density maps. The method is based upon ATTRACT, an atom-based protein-protein docking program [51], [52], which has been recently extended to deal with multiple molecules and with symmetry (De Vries and Zacharias, to be published). Unique among docking programs, ATTRACT uses a coarse-grained forcefield, where proteins are represented by up to 4 (pseudo-)atoms per amino acid. Building upon this, an atom-to-grid cryo-EM fitting protocol was developed, using ATTRACT's coarse-grained atom model and energy minimizer to assemble all components simultaneously into the electron density map. Initial models are fitted using low-resolution data and then re-scored using a novel gradient vector matching algorithm. Finally, the best models are refined with a higher resolution density map, using the ATTRACT force field to optimize the interfaces.

ATTRACT-EM aims to cover the middle ground between rigid fitting methods and flexible fitting methods, in terms of speed, accuracy and level of detail. Therefore, ATTRACT-EM could be applicable in scenarios where cryo-EM alone does not provide the answer, and additional sources of information are available. In addition, although we did not investigate this possibility in the current study, the ATTRACT program offers normal-mode based flexibility, which can be added to the assembly protocol during or after the initial assembly stage. ATTRACT-EM is aimed specifically at large molecular machines, where methodological improvements regarding *in silico* assembly would be the most beneficial in providing new biological insights.

In order to assess the performance of ATTRACT-EM, it was applied to the GroES-GroEL complex and a number of smaller complexes. Successful application to the test systems required the inclusion of experimental information on the symmetry of the systems, or, in case of small dimeric complexes, the approximate location of the subunits in the electron density. For the GroES-GroEL cis ring (using a simulated electron density), the best-ranked ATTRACT-EM model had an RMSD of only 2.4 Å from the crystal structure. For the entire GroES-GroEL complex, with experimental electron density data, the best-ranked model had an RMSD of 4.6 Å, and a 2.8 Å model was ranked in the top 10.

This is a significant improvement over existing methods for simultaneous rigid fitting, and was possible mainly through a new method, gradient vector matching (GVM), for scoring the agreement between model and density map.

## Materials and Methods

### General assembly protocol

**Figure 1:**
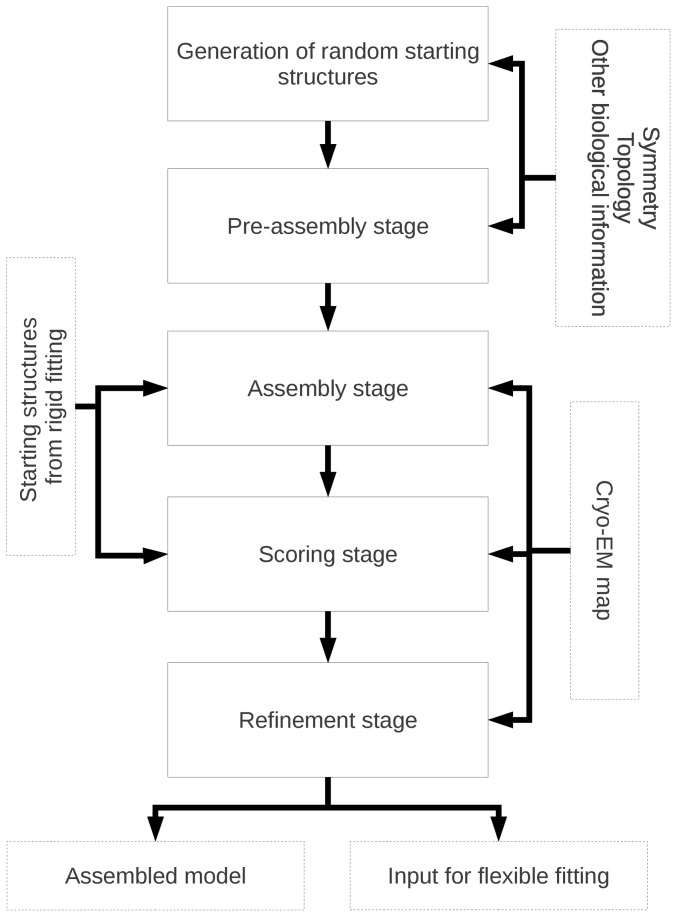
Flowchart of the ATTRACT-EM protocol.

The ATTRACT-EM assembly protocol consists of the following five stages. Stage 2, 3 and 5a–5d are energy minimization stages. Details of the methodology of the different stages of the protocol are discussed in the rest of the section. A flowchart of the protocol is shown in figure 1.

1. **Generation of the starting structures (typically 1 million).** The most straightforward way to do this is to generate positions and orientations for each of the components randomly. However, ATTRACT-EM can start from any structure. Therefore, it is possible to use some biological information in the generation of starting structures, for example symmetry or approximate location. Finally, the structures could also come from an external source, e.g. the output of a rigid fitting program.
2. **Pre-assembly stage.** This stage is meant to impose symmetry upon random starting structures, and can be skipped. All atomic and cryo-EM forces are turned off. The symmetry penalty is minimized, while either the position of the structures is kept fixed, or they experience weak distance restraints to keep them at a reasonable distance from each other. Typically, 500 000 structures are selected after this stage.
3. **Assembly stage.** All components are assembled into the electron density map using a Gaussian overlap model. The density map is down-sampled to low resolution (typically 40 Å), making the Gaussians long-range and smooth. In addition, a weak center-of-mass distance restraint (“gravity”) is added to further facilitate the assembly process. Finally, a “voxel atom density” energy term is added to prevent excessive overlap between the components. Atomic forces are still turned off.
4. **Scoring (re-ranking) stage.** First, the structures are sorted according to the final score from the assembly stage and the top 100 000 structures are taken. Those structures are then sorted according to Gradient Vector Matching (GVM) energy, using the original density map (20 Å or 23.5 Å in the current study)
5. **Refinement stage.** The top scoring structures (typically 1000) are subjected to a multi-stage refinement, each stage consisting of re-minimization of the structure.
  - An energy term for the original density map is added
  - Atomic forces (from the ATTRACT force field) are turned on, with a weight of 0.01.
  - Atomic forces are set to full weight. To prevent disintegration of the structure, the cryo-EM density overlap terms are scaled with a weight of 50.
  - Both atomic forces and EM density overlap have their weights set to 1

After the refinement stage, the final structures are scored and re-ranked according to their GVM energy.

### Gaussian overlap model

ATTRACT-EM is based on the idea that each atom should have a degree of overlap with the cryo-EM map, or else should experience an attractive force into the map. In ATTRACT-EM, each (coarse-grained) atom of each component is represented by a Gaussian. The experimental (or simulated) cryo-EM map is represented as well as a set of Gaussians, positioned at the center of each voxel of the map. To obtain this representation, a deconvolution algorithm was applied to the original density map, using the Clarity deconvolution library [61].

The advantage of this representation is that the overlap O between two Gaussians is itself a Gaussian, which can be computed efficiently and independently of orientation:
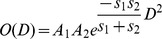
where D is the distance between the Gaussians, A_1_ and A_2_ are the amplitudes of the Gaussians, and s_1_ and s_2_ are decay factors derived from the standard deviations 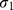 and 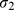 of the Gaussians: 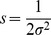


Here, we choose the standard deviations of all Gaussians, both atomic and voxel Gaussians, to be half the resolution of the cryo-EM density map, divided by 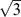 for the three Cartesian dimensions.

The derivative of this function is then as follows:
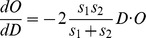
This derivative function is computed in each of the Cartesian dimensions X, Y and Z, which is required for energy minimization.

The next step is to compute the maximum possible overlap that an atom can experience. Any atom that experiences less than this overlap should then experience a force, with its direction defined by the Cartesian derivative computed above. The magnitude of the force was defined to be linearly proportional to the deficiency in overlap (harmonic potential).

Unfortunately, the computation of the maximum overlap is not trivial. If we were fitting one atom to a density map of one atom, the maximum overlap would be simply 1. However, for a multi-atom assembly the maximum overlap will be much more: since the distance between atoms is much smaller than the resolution, there is considerable “spillover” overlap from neighbouring atoms, in particular for atoms in the core. Therefore, for each protein component, the maximum overlap values were determined by computing the overlap of the component with itself. To do this, a simulated density map was generated for the component, which was then deconvoluted. For each atom, the actual overlap with this map was computed, and this value was considered the maximum possible overlap for this atom. These maximum overlap values for all components were concatenated into a single file.

However, these per-component maximum overlap values are correct only if a single component, accounting for the complete electron density, is fitted into the map. Although the introduction of flexibility may cause some inaccuracies in these values, these inaccuracies should be limited as long as one deviates not too much from the starting conformation. In contrast, for multi-component cases, atoms experience significant spillover from a different source, coming this time from adjacent subunits (intermolecular spillover; in addition to the intramolecular spillover accounted for above). The actual amount of intermolecular spillover is dependent on the resolution of the map, and also of the compactness and arrangement of the subunits in the map. In this study, for simplicity, we chose to model this spillover as a single parameter that scales the intramolecular maximum overlap with a factor F. To determine F, the reference structure was inspected and the value was estimated where the restraint violation energy started to rise sharply. For the GroEL cis ring, F was found to be 5.1 (for the 40 Å map; F was 1.7 for the 20 Å map); for the full GroES-GroEL complex, the factor F was found to be 7.0 (for the 40 Å map). Finally, for the experimental GroES-GroEL map, F was found to be 9.8 (for the 44.8 Å map). We are fully aware that in a real-world situation, F is not known, and that in any case a single factor is a too simplistic model: in reality, inter-component spillover will vary between components and between regions within a component. Therefore, estimation of the maximum expected overlap is something that should be properly optimized in each situation, based on a priori knowledge on topology, radius of gyration, and knowledge on which regions of a subunit interact with other subunits (hence receiving a higher intermolecular spillover). However, this is only necessary for low-resolution data. In contrast, density maps with a resolution that is much better than the size of a single component will not experience much intermolecular spillover, and here F can be set to 1.

In ATTRACT-EM, in addition to achieving an overlap of 100% of the maximum overlap with F = 1 (with a large violation energy penalty if this overlap is not achieved), the structure also has to match an overlap that was multiplied by F as estimated above, associated with a much smaller energy penalty.

Note that the Gaussian overlap function and the F parameter are only used during the assembly and refinement stages, not during the scoring (re-ranking) stage, when only the GVM energy is used (see below).

### Pre-computation

At runtime, the overlap of an atom can be computed by summing the overlap of an atom with each voxel (or, to be more exact, voxel-centered Gaussian) of the electron density map. For this, we expected it to be sufficient to take into account only the nearest voxels. However, this turned out to be false, with far voxels providing a non-negligible contribution to the overlap (results not shown); taking this into account severely hampered the speed of the overlap function. Therefore, a pre-computing step was introduced. For each voxel in the density map, the overlap (and its gradient) was computed between a hypothetical atom, placed at the center of the voxel, and the entire density map. During the fitting, the overlap of an atom was computed by trilinear interpolation: the eight neighboring voxels of an atom were determined and each voxel's pre-computed overlap was weighted proportionally in approximating the total overlap. This effectively “divides” the atom fractionally among all eight neighboring voxels, with relative weights inversely proportional to the distance. Due to the smoothness of the Gaussian function, and its flat plateau near the center, this approximation is extremely accurate (results not shown).

### Voxel atom density

A final issue in the simultaneous fitting of multiple components is the fact that the components should not clash or overlap with one another. To prevent this, we introduced a simple per-voxel atom density term. Since the fitting algorithm already “divides” each atom among the neighboring voxels (see above), these atom weights are simply accumulated during the fitting process, measuring the “voxel atom density”, i.e. how many atoms are present in a voxel. These densities were then compared with standard maximum atom densities, and a repulsive force was applied to all atoms in a voxel were the atom density was too high. The standard densities were determined from the reference complex of the cis ring (18 for 40 Å maps, lowered to 16 during refinement; 4 for 20 Å maps), but they are expected to be universal for folded proteins with normal packing (for a given voxel size). To verify this, we used the average protein packing densities of Tsai et al. [62] and the average weight of an ATTRACT pseudo-atom to calculate the standard atom density value (see Supporting Analysis 6 in Supporting File S1). The standard voxel atom density was calculated to be 14 per voxel on average for the protein interior, with some fluctuations. Our value of 16 (and 18 during the assembly stage) accounts for these fluctuations (and allows for limited clashes between the components in the assembly stage).

For the simulated GroES-GroEL complex, the same maximum density values were used. For the experimental map, slightly larger values were used in accordance with the larger voxel size (19 for the 44.8 Å map, 4.4 for the 23.5 Å map). Several small control complexes exhibited abnormal packing (see Supporting Analysis 1 in Supporting File S1) and the maximum voxel densities were increased to improve the sampling.

### Gradient Vector Matching (GVM) energy

For every voxel 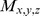 in a grid, a gradient *V* can be defined that captures the difference in electron density with its neighbors. In principle, such a gradient can be computed with the Laplacian filter method as follows:
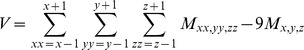
However, unlike the Laplacian filter, which aims only to capture only the *magnitude* of curvature, we aimed to capture the *direction* of the density gradient as well. In other words, the aim is to compute *V* as a Cartesian gradient vector, which can be done in the following manner:
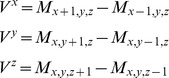
However, since this formula no longer averages over multiple voxels, it is even more sensitive to noise than a Laplacian filter. Therefore, to reduce the noise, the surrounding voxels were taken into account for the calculation of V, leading to the following final formula, which is also known as the 3D Prewitt operator:
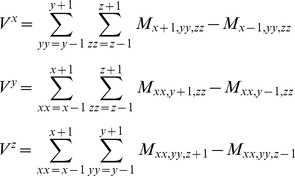
During the GVM scoring, this formula was then applied to two different grids M, resulting in two different assessments of *V* for each voxel. First, the experimental gradient vector (*V*_exp_) was computed with M being the experimental (or simulated) electron density map at 20 Å (or 23.5 Å) resolution. Then, the model gradient vector (*V*_model_) was computed with M being the grid of voxel atom densities, i.e. the summated fractional atom occupancy of each voxel. The *V*_model_ vectors were found to be systematically smaller by a factor of about 1.35 than those derived from electron density, and were scaled accordingly.

The deviation between *V*_exp_ and *V*_model_ was converted into an energy term. For all voxels with a significant magnitude of *V*_exp_, the difference between *V*_exp_ and *V*_model_ was computed. The threshold for significant magnitude was chosen to be 2 units throughout the study. For the cis ring, for each xyz component, a deviation of 2 units was tolerated, but further deviation was penalized with an energy proportional to the square of the deviation. For later experiments with the entire GroES-GroEL complex, for which the gradients were larger and much more numerous, the tolerance threshold was increased to 20.

### System

The method was tested on the GroES-GroEL complex (PDB code 1AON [34]). This assembly consists of 21 components, organized in three symmetric, homoheptameric rings: GroES (chain O-N), GroEL cis (chain A-G) and GroEL trans (chain H-N). During fitting, seven copies of chains A, O and/or N were assembled into the electron density: generated models were compared to a reference structure, which was built by fitting coordinates of each of the three chains seven times onto the coordinates of 1AON using PyMol [63]. Since there are minor differences between the intra-ring components, there is a minor RMSD of 0.4 Å (C-alpha RMSD) between the reference structure and 1AON.

In addition, C7 symmetry restraints were imposed in the form of distance restraints (see below). Since the symmetry in 1AON is non-crystallographic and not completely perfect, even the reference structure had a small symmetry violation energy, but this did not interfere with the fitting process.

An experimental cryo-EM density map for GroES-GroEL was taken from the EM databank (code 1046, resolution 23.5 Å). Adaptation of this map to ATTRACT-EM, however, presented a number of complications. First, all our simulated maps had a resolution-to-voxel ratio of 4, and we discovered that the assembly and in particular the GVM scoring do not work well otherwise. In contrast, the experimental map has a voxel size of 2.8 Å at a resolution of 23.5, which is a ratio of about 8. Therefore, neighbouring voxels in the original experimental map were joined, doubling the voxel size to 5.6 Å, and this processed map was used in lieu of the 20 Å map in the simulated run. Repeating this procedure yielded voxels of 11.2 Å, and the resulting map was filtered down to 44.8 Å resolution using the EMAN program [64], [65]. This second processed map was used in the initial fitting, in lieu of the 40 Å map in the simulated run. Both maps were rescaled to have the same density sum values as the combined simulated maps of the components.

As with the simulated maps in the previous runs, a Gaussian deconvolution was performed on the two experimental maps. However, the noise in these maps produced so many deconvolution artifacts that the deconvolution step was omitted and the non-deconvoluted maps were used instead. It is to be investigated if this omission of the deconvolution step is to be used with experimental maps in general or if it is specific to the EMD-1046 map.

Finally, since the experimental EMD-1046 map is in a different coordinate frame than 1AON, a transformation matrix had to be determined. This matrix was obtained by fitting the reference structure to 1GRU, the fitted structure provided by the authors of the GroES-GroEL density map [35]. This transformation matrix was applied to each component prior to fitting with experimental data, and the reverse transformation was applied afterwards.

### Generation of starting structures for the full GroES-GroEL complex

Basic information from the experimental cryo-EM density map (EMD code 1046) (Figure S1 in Supporting Information File S1) was used in the generation of starting structures for the full GroES-GroEL map.

Figure S1 shows clearly that GroES-GroEL has the form of an elongated igloo, consisting of two hollow rings (the GroEL rings) covered by a smaller cap. The axis of symmetry is easily determined; in fact, the experimental cryo-EM map has the symmetry axis already aligned to the Z axis.

In the reference structure, the three rings (GroES, GroEL cis and GroEL trans) have a Z position of −64.4 Å+/−0.2, 0.0 Å+/−0.1, and 71.4 Å+/−0.1, respectively (average position of the center of mass of the seven components of each ring). In the starting conformations, each component was placed at this Z position with a random displacement of −5 to 5 Å (the same displacement for each component within a ring, different displacements per ring and per structure).

In the reference structure, the three rings have a radius in the xy plane of 26.6 Å+/−0.4, 46.0 Å+/−0.3 and 46.8 Å+/−0.4, respectively. For the generation of starting conformations, for each of the three rings, a random radius of 35 Å+/−15 was chosen. The x and y coordinates of each ring's first component were chosen at random and scaled to conform to the chosen radius; the other components were then arranged forming an evenly spaced circle in the xy plane. Finally, each component was oriented at random.

These radius and random component parameters were all initial guesses and not optimized in any way. As usual, 1 million structures were generated and subjected to a pre-assembly minimization stage to impose symmetry; however, since the symmetry was already perfect in terms of position, positions were fixed and only the orientation was minimized. As usual, the structures were ranked according to symmetry energy and the best 500 000 structures were subjected to the assembly stage.

### Symmetry restraints

C7 symmetry was imposed upon each of the three rings in the form of harmonic distance equality restraints, using the same algorithm as the docking program HADDOCK [46], [48], and also similar to the distance restraints used in GMFit [29]. This algorithm does not require the explicit definition of symmetry axes. The algorithm was implemented and generalized to enforce C_X_ symmetry distance restraints for any number of monomers X.

First, all C-alpha atoms *C* are arranged into pairs, each pair containing atom *A* = *C_i_* and atom *B* = *C_p−i_*, where *p* is the total number of C-alpha atoms in a monomer, and *i* iterates over all positive integers where *i*≤*p−i*. Then, for each atom *A* and *B* in a pair, one or more equalities are imposed. Equality *S_k_* is defined as *d*(*A_n_*, *B_n+k_*)≡*d*(*A_n+1_*, *B_n+k+1_*)≡*d*(*A_n+2_*, *B_n+k+2_*) …, where *n* iterates over X, and *d*(*A_j1_*, *B_j2_*) is the distance between atom *A* in monomer *j1* and *B* in monomer *j2*. *j1* and *j2* are modulo monomer indices, i.e. if X = 7, *A_8_*≡*A_1_*. *k* is an integer whose allowed values depend on X, with 0<*k*<½ X.

Thus, for C7 symmetry, for each pair, three equalities *S_1_*, *S_2_* and *S_3_* are imposed, with *S_1_* defined as *d*(*A_1_*, *B_2_*)≡*d*(*A_2_*, *B_3_*)≡*d*(*A_3_*, *B_4_*)≡*d*(*A_4_*, *B_5_*)≡*d*(*A_5_*, *B_6_*)≡*d*(*A_6_*, *B_7_*)≡*d*(*A_7_*, *B_1_*).

We also implemented D4 restraints (for the tetramer 7CAT, see Supporting Analysis 1 in Supporting File S1), using the three distance equalities *d*(*A_1_*, *B_2_*)≡*d*(*A_3_*, *B_4_*) ; *d*(*A_1_*, *B_3_*)≡*d*(*A_2_*, *B_4_*); *d*(*A_1_*, *B_4_*)≡*d*(*A_2_*, *B_3_*).

Each equality is enforced through a harmonic distance restraint on each of the distances in the equality distance set. First, the average distance in the set is computed, and for each distance in the set, the deviation D is computed. A harmonic force F = − c * D and the corresponding energy E = 2 * c * D are then computed, with a force constant c = 0.005 kcal/mol/Å. While this force constant is small, the number of harmonic restraints scales both with the number of components (quadratically) and the number of C-alpha atoms (linearly), causing several thousands of restraints to be defined, even for just the GroEL cis ring.

### Location restraints

For each monomer in each of the assembled structures, three random offsets were chosen, between −5 and 5 Å. These offsets were added to the XYZ components of the true location, leading to an average displacement of 4.8 Å. During minimization, the center-of-mass position was restrained to this location using a harmonic potential with a force constant of 0.5 kcal/mol/Å.

### RMSD calculations

The overall Root Mean Square Deviation (RMSD) was simply computed as the RMSD between all heavy atoms in the model and the reference structure, without any coordinate fitting or optimization. In addition, the ligand RMSD between adjacent components was computed, measuring not the absolute but the relative positioning of the components. For each pair of adjacent components, the coordinates were extracted from both the model and the reference structure, and the ligand RMSD was computed in the standard way [54], [55]: the model coordinates of the first component (the “receptor”) were fitted onto the reference, and for the second component (the “ligand”), the RMSD between the model and reference coordinates was computed. Reported ligand RMSD values are the average of the ligand RMSDs of all seven equivalent component pairs.

## Results

### Method

Our aim is to develop a cryo-EM fitting function that can be added as an energy term to ATTRACT, acting in synergy with the existing intermolecular energies (van der Waals, electrostatic and symmetry energies). This allows the fitting to be optimized using ATTRACT's existing energy minimizer. It also allows a multi-stage protocol: first, the initial fitting is performed with low resolution cryo-EM data (sampling/searching); then, gradually more detail is added, in the form of atomic intermolecular forces and higher resolution cryo-EM data, in order to refine the results and to discriminate correct from incorrect solutions (re-ranking/scoring). This subdivision of the problem into sampling, ranking (scoring) and refinement is common in the protein docking field and has proven its merit [66].

However, this does impose a number of requirements on the cryo-EM fitting function. First, it must be described in terms of energies and forces that act on individual atoms. Second, even in simple two-body docking, energy minimization is repeated tens of thousands of times with different initial conformations, and this number must be vastly greater for large assemblies; therefore, the function should be computed relatively fast. Third, the function must not get trapped easily in local energy minima, so forces should be long-range and smooth. Finally, it is not our aim to develop a global cryo-EM fitting method, which attempts to maximize the overlap with the electron density; rather, the fitting function should restrict the conformational space that is available to the ATTRACT docking program. Therefore, it should not be overly restrictive, allowing components to move around within regions of high electron density, so that they can adapt to each other by forces of symmetry and ATTRACT intermolecular energies. This also means that the fitting function should be *local*, taking into account the local electron density environment of each component, rather than the overall global fit; as a beneficial side effect, a local fitting function also allows the sum of all components to be smaller than the cryo-EM map.

A fitting function fulfilling the requirements above was developed and coded into ATTRACT, based on the overlap between Gaussians (see Materials and Methods). To avoid any reliance on clustering or feature detection, each individual atom of the protein and each individual voxel of the density map is represented by a separate Gaussian. We found that at 40 Å resolution, the Gaussian overlap function is very smooth and long-range, enough to pull the components into the map. During the fitting, each atom is “divided” (with proportional weights) among the eight closest voxels in the electron density grid. This allows us to pre-calculate all possible atom-grid interactions, which increases both speed and accuracy (see Materials and Methods). It also allows to keep track of the “voxel atom density”, i.e. the amount of atoms that are present in any given grid voxel. By introducing an additional energy term that penalizes excessive voxel atom densities, interpenetration between the components is prevented. This is because during the initial fitting stage, the ATTRACT force field is disabled to smoothen the overall energy landscape.

This Gaussian overlap function and voxel atom density function were used in all computational assembly experiments performed in this study.

### Application to the GroES-GroEL cis ring

For an initial test, the method was applied to the homoheptameric GroEL cis ring. The ring was isolated from 1AON and the coordinates were centered. Simulated density maps of 20 Å and 40 Å were generated from the coordinates using the Situs pdb2vol tool [6]. The seven components were then assembled into the density map by energy minimization using ATTRACT, in three stages. First, starting conformations were generated by placing each component at a random position and orientation. Then, a pre-assembly stage was used to impose symmetry onto the starting conformations. Note that the symmetry only restrains the relative orientations of the monomers, not their absolute positioning. To allow the necessary rearrangements to take place, all other forces were turned off and the proteins were allowed to move freely through one another; however, a weak, long-range attractive force and a strong, short-range repulsive force were applied between the centers of mass in order to preserve a reasonable distance between the components. The pre-assembly stage is shown in Supporting Information Movie S1.

Finally, out of the 1 million generated structures, 500 000 structures with the lowest symmetry violation energy were selected and subjected to the actual assembly stage. In the assembly stage, each atom of each component was fitted into the 40 Å simulated cryo-EM map using the Gaussian overlap paradigm described above, using voxel atom density to prevent major clashes between the components. The ATTRACT intermolecular forces were still disabled, but symmetry restraints were maintained during the fitting process: therefore, the minimized energy is the sum of the density fitting energy towards the 40 Å map, and the symmetry energy. The assembly stage is shown in Supporting Information Movie S2.

**Figure 2:**
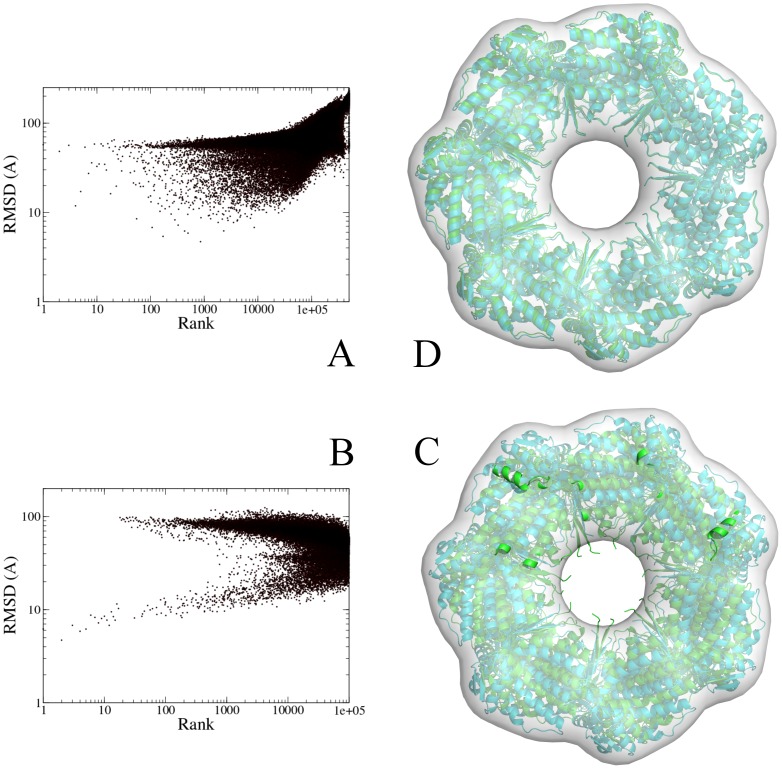
Assembly results for the GroEL cis ring. A) RMSD values of all generated models compared to the reference structure; models were fitted (energy-minimized) using a 40 Å simulated density map and ranked by the same energy. B) RMSD values of the generated models compared to the reference structure; the top 20% models from A) were rescored with GVM using a 20 Å simulated density map. C) Overlay of the best-scoring structure from B) (green) on the reference structure (cyan) (RMSD: 5.4 Å). Image was generated with PyMol [63]. D) Overlay of the best-scoring refined structure (green) on the reference structure (cyan) (RMSD: 2.5 Å). Structures from C) were refined using the ATTRACT force field and rescored with GVM. Image was generated with PyMol [63].

Figure 2a shows the results of the initial assembly stage, with the models ranked by the total energy, and the rank plotted against the RMSD towards the reference structure. Figure 2a shows that a 40 Å map is in principle enough to generate models with a low RMSD, although not many of these structures are generated: only 37 out of 500 000 structured had an RMSD better than 10 Å. In addition, it places virtually all of those structures (35/37) in the top 10 000 (top 2%) and many of them (11/37) even in the top 1000, including the structure with the best RMSD (4.7 Å RMSD). Table 1 shows the RMSD values of the top 10 models and also their ligand RMSD values, which describes their relative positioning. However, none of these structures have an RMSD under 10 Å, although the 4^th^ structure comes close.

**Table 1:** Top 10 RMSD values for the GroEL cis ring after the assembly stage.

Rank	Overall RMSD	ligand RMSD
1	57.4	58.3
2	48.3	33.5
3	56.5	68.1
4	11.9	8.4
5	17.3	16.6
6	53.8	64.2
7	27.4	57.0
8	36.0	49.9
9	58.2	35.6
10	32.8	54.3

RMSD and ligand RMSD values of the top 10 models for the GroEL cis ring (in Å), compared to the reference structure; models were fitted (energy-minimized) using a 40 Å simulated density map and ranked by the same energy.

The 20 Å density map was deliberately not used in the initial assembly stage: while its higher resolution provides somewhat more details, it is also less smooth and therefore less potent in pulling components into the map from a long range. To confirm this, a comparison run was performed where the 20 Å map was used already in the initial assembly stage, which led to much poorer RMSD values (results not shown). In addition, refinement with the 20 Å density map led to somewhat improved solutions, but still with wrong solutions at the top (see Supporting Analysis 2 in Supporting File S1). Clearly, our implementation of the Gaussian-overlap fitting function (in combination with the ATTRACT energy minimizer) is suitable for the sampling of correct solutions; however, when it comes to scoring and ranking, the function is unable to fully distinguish correct solutions from wrong ones.

**Figure 3:**
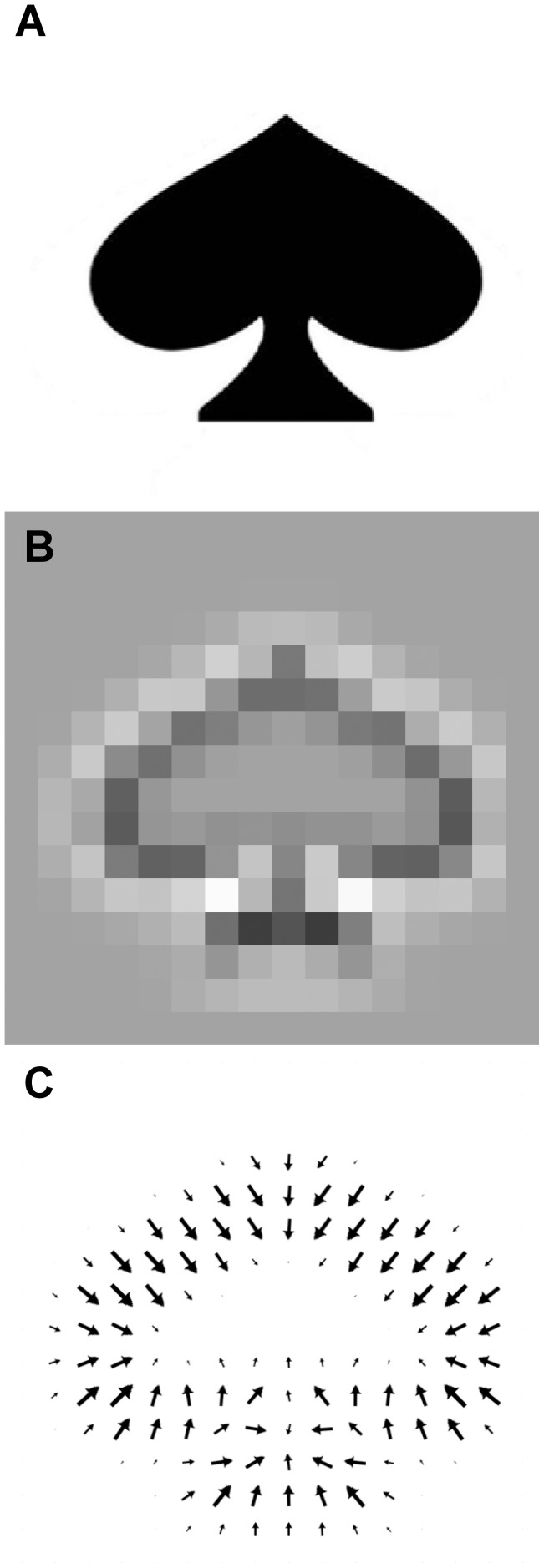
The gradient vector representation. A) A simple reference shape. B) The reference shape with a Laplacian filter applied to it. C) The gradient vector representation of the reference shape. The direction of the arrow indicates the direction of the gradient vector, whereas the size of the arrow shows its magnitude.

It occurred to us that the Gaussian-overlap fitting algorithm does not completely grasp the features of the density map. It is easy to see that the wrong solutions do not fit the map, because the contours of the map are very different from that of the model: however, the Gaussian-overlap function only takes into account the values of the density map voxels. Chacon and Wriggers [20] developed a Laplacian filter to emphasize the contours of a density map, interpreting “contours” as the regions where the electron density undergoes steep changes (i.e. with high magnitude of its second derivative). However, the Laplacian filter may be sensitive to high-resolution noise [20], [67], and it computes only the location and magnitude of a contour, not its orientation. In addition, it was designed for grid-to-grid fitting whereas our method performs atom-to-grid fitting. Therefore, we developed a different contour-matching algorithm, which we dub “gradient vector matching” (GVM), with several modifications to the Laplacian filter of Chacon and Wriggers. We reasoned that for an atomic representation, the contours are where the protein surface starts, i.e. where the number of atoms in a certain region suddenly changes. Since the fitting procedure already computes the voxel atom density of the model, we decided to use these values and compare them to the actual EM density values of the map. Second, we wanted to calculate not only the magnitude of changes in atom density/electron density, but also their direction: in this, we interpret a contour as a polygon, or part of a contour plane, where the gradient vector of the electron density/atom density corresponds to the contour plane's normal vector, defining its orientation in space. This vector can be computed by taking the difference between neighbouring voxels in the X, Y and Z directions (3D Prewitt operator; see Materials and Methods). This vector interpretation of contours is conceptually (but not computationally) similar to the normal/gradient vectors in the 3SOM fitting method [23] and in the MOTIF-EM superposition tool [68]. The gradient vector representation is shown in two dimensions in figure 3.

Supporting Analysis 3 in Supporting File S1 shows the correlation between computed and experimental gradient vectors. The mismatch between the two was converted to an energy term (see Materials and Methods).

Figure 2b and table 2 show the RMSD statistics for all the initial models sorted by GVM energy. Despite the fact that only 37 out of 200 000 structures had an RMSD<10 Å, the entire top 10 consists of these structures, and all of them were scored in the top 1000. Moreover, there were only 7 structures with RMSD<7 Å, and these were ranked 1–6 and 13. The top scored model has an RMSD of only 5.4 Å, and figure 2c shows an overlay of this model with the reference structure.

**Table 2:** Top 10 RMSD values for the GroEL cis ring after the scoring stage.

Rank	Overall RMSD	ligand RMSD
1	5.4	6.3
2	6.2	13.1
3	4.7	7.9
4	6.8	10.5
5	5.9	11.2
6	6.2	7.7
7	8.7	7.7
8	8.3	17.1
9	7.2	13.6
10	8.0	14.9

RMSD and ligand RMSD values of the top 10 models for the GroEL cis ring (in Å), compared to the reference structure; models were fitted (energy-minimized) using a 40 Å simulated density map and the top 20% structures were ranked by gradient matching using a 20 Å simulated density map.

### Molecular refinement

In order to further improve the accuracy of the models, a multi-stage refinement procedure was employed. First, the 20 Å map was added to the fitting energy and the top structures were re-minimized and re-ranked using GVM energy. A large gap in energy was observed between the first 32 structures (GVM energy: 44–2164) and the next structure (GVM energy: 17 413). These first 32 structures, with RMSD values between 2.9 and 10.7 Å, were selected and refined using the ATTRACT intermolecular force field. In the first stage, the intermolecular forces were turned on, but scaled by a factor of 0.01. In the next stage, full intermolecular forces were used but the fitting energy was scaled up by a factor 50 to prevent the structure from disassembling. In the final stage, both intermolecular and fitting energies were scaled normally. The final structures were then scored and re-ranked according to the GVM energy, without any weighting scheme. The molecular refinement is shown in Supporting Information Movie S3.

Table 3 shows the result of the refinement procedure. The top ranked model is highly accurate, showing an overall RMSD of only 2.5 Å, with interfaces that are essentially perfect (ligand RMSD 1.0 Å). Figure 2d shows the overlay between this model and the reference structure. In addition to having the best GVM score, this structure also had the most favored ATTRACT force field energy.

**Table 3:** Top 10 RMSD values for the GroEL cis ring after the refinement stage.

Rank	Overall RMSD	ligand RMSD
1	2.5	1.0
2	6.2	7.5
3	6.6	4.8
4	7.2	11.1
5	6.2	9.8
6	8.1	12.5
7	7.1	11.0
8	7.1	1.0
9	7.2	7.5
10	7.8	4.8

RMSD and ligand RMSD values of the top 10 models for the GroEL cis ring (in Å), compared to the reference structure; models were fitted (energy-minimized) using a 40 Å simulated density map, ranked by gradient matching using a 20 Å simulated density map, and refined using the ATTRACT force field.

In general, the molecular refinement did not much affect the overall RMSDs, but ligand RMSDs showed a dramatic improvement. In fact, in several cases (for example the structure with rank #8), the strong intermolecular forces caused the components to shift within the ring plane, leading to a degradation of overall RMSD even while the interfaces were assembled perfectly.

The 2.5 Å RMSD of the top-ranked model is a large improvement over previous attempts of Zhang et al. [31], who also assembled the cis ring using their IQP method, achieving an RMSD of 8.6 Å. In contrast, our method achieves an RMSD of 5.4 Å after initial fitting and scoring, and 2.5 Å after refinement. For a fair comparison, it must be mentioned that Zhang et al. used a segment of the experimental EMD-1046 density map. Simulated data was used in our study to exclude any possible artifacts arising from incomplete separation of the rings at low resolution.

### Application to the full GroES-GroEL, simulated data

Next, the method was tested on the full GroES-GroEL complex, assembling 21 components simultaneously. Again, 20 Å and 40 Å electron density maps were simulated using Situs, and random starting structures were generated as before, using the seven-fold symmetry of each ring as distance restraints. However, we found that for 21 components, brute force fitting of random starting conformations did not produce any structures of good RMSD, not even when the number of structures was increased by an order of magnitude (results not shown). Therefore, the three-ring topology and symmetry axis were used in the generation of starting structures (see Materials and Methods). The GroES-GroEL system serves as a test system to evaluate the approach, and one may argue that such information may not be available in other cases. For GroES-GroEL, however, the approximate locations of the rings are evident from the cryo-EM map, as shown by figure S1 in Supporting Information S1. In fact, the approximate location of the rings had been inferred from negative stain EM already long before the X-ray or cryo-EM structure of the assembly became available [69]. No absolute positions or any other information from the crystal structure were used.

The results of the initial fitting were similar to those for the cis ring alone, except that sampling was worse: out of 500 000 structures, only five had an RMSD under 10 Å (Table S2 in Supporting Information File S1).

**Figure 4:**
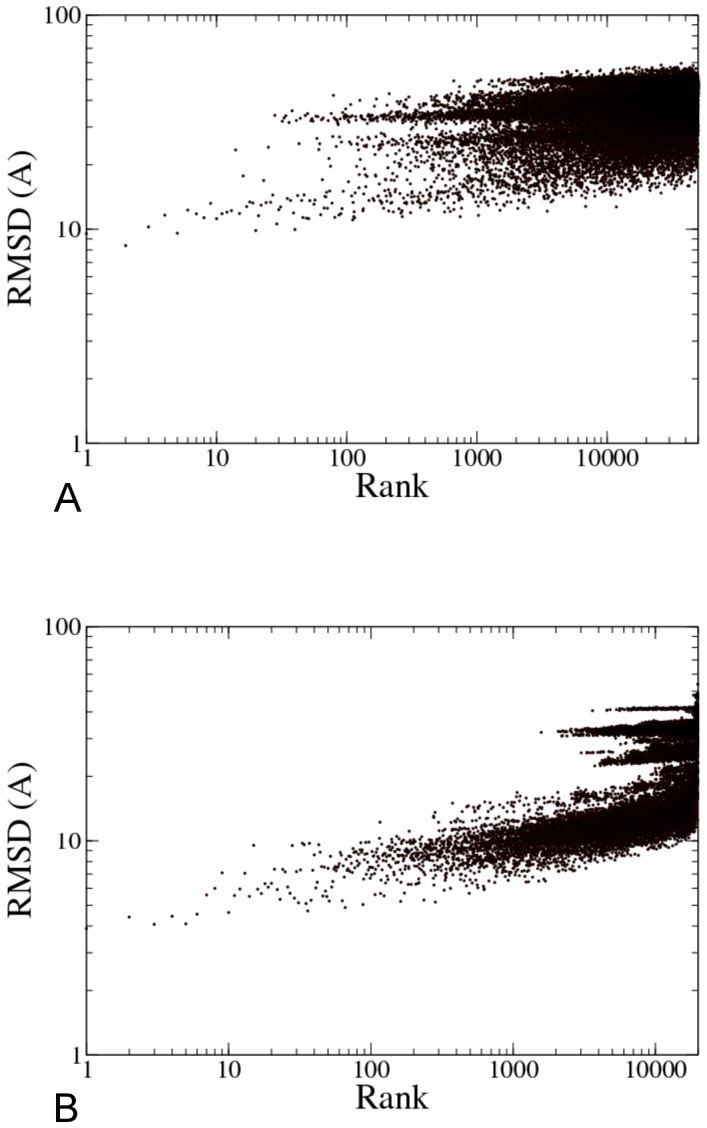
Assembly results for the full GroES-GroEL complex, using simulated data. A) RMSD values of generated models for the full GroES-GroEL complex, compared to the reference structure; models were assembled using a 40 Å simulated density map and rescored with GVM using a 20 Å simulated density map. B) RMSD values of recombined models for the full GroES-GroEL complex, compared to the reference structure; the top 100 models from A) were subjected to ring recombination; the resulting combinations were rescored with GVM.

Figure 4a shows the ranking of the generated structures by GVM energy. This shows that GVM works equally well for the full GroES-GroEL complex as for the cis ring. Three of the structures under 10 Å are in the top 10, including the best generated structure (RMSD 8.4 Å, rank #3).

RMSDs were also calculated separately for each of the three rings (table 4). This shows that the RMSDs of the cis ring are in fact similar to the previous computational assembly experiment; the trans ring, too, has low RMSDs similar to the cis ring, but the small GroES ring is placed very poorly. Still, several structures with a correct GroES ring were generated; however, in none of these structures, both rings of GroEL were correctly placed as well. Due to the small size and smooth features of the GroES density, structures with good GroEL placement but poor GroES placements were favored by the GVM scoring over structures where the opposite was the case. Still, we hoped that the GVM would be able to pick out structures where all three rings were placed correctly, so we sought to improve the sampling in order to generate such structures.

**Table 4:** Top 10 RMSD values for GroES-GroEL with simulated data, after the scoring stage.

	Overall RMSD			
			GroEL	GroEL
rank	all	GroES	cis	trans
1	10.8	33.5	5.5	3.8
2	9.5	26.4	5.0	6.5
3	8.4	15.2	7.6	7.2
4	10.2	18.5	6.0	11.2
5	11.6	23.7	12.2	6.5
6	9.6	21.8	6.9	7.8
7	12.3	23.9	8.1	12.5
8	11.8	24.3	12.1	6.7
9	11.3	27.1	8.3	8.5
10	13.2	30.7	9.3	8.4

RMSD values of the top 10 models for the full GroES-GroEL complex (in Å), compared to the reference structure; models were fitted (energy-minimized) using a 40 Å simulated density map and ranked by gradient matching using a 20 Å simulated density map.

Apart from the intra-ring contacts, which are symmetrical and therefore highly interdependent, GroES-GroEL contains two interfaces that are independent from another: GroES/GroEL-cis and GroEL-cis/GroEL-trans. Therefore, it was decided to take the top 100 structures and recombine them, iterating over all possible pairs of structures and taking two adjacent rings from one structure and the third ring from the other.

This resulted in 19 800 recombined structures, which were pooled with the 100 original ones, and re-scored and re-ranked according to the GVM energy. Figure 4b and table 5 show that the sampling is indeed much improved. Moreover, GVM is able to select the correct structures: the structures in the top 10 have excellent RMSD values, with the top-ranked structure at 4.0 Å. The rings are assembled correctly, not only regarding their absolute position in the map, but also the relative positioning of the components within the ring. Most of the residual error comes from the inter-ring positioning, in particular between GroES and cis-GroEL. This is not surprising since the interface between GroES and cis-GroEL is very small (825 Å^2^ per component, versus 3226 Å^2^ between adjacent cis-GroEL components).

**Table 5:** Top 10 RMSD values for GroES-GroEL with simulated data, after ring recombination.

					ligand RMSD				
	Overall RMSD				intra-ring			inter-ring	
			GroEL	GroEL		GroEL	GroEL	cis-	cis-
Rank	all	GroES	cis	trans	GroES	cis	trans	GroES	trans
1	4.0	4.3	3.9	3.9	4.2	7.1	5.2	9.8	7.5
2	3.9	4.3	3.9	3.8	4.2	7.1	7.7	9.8	7.3
3	4.4	4.3	3.9	4.9	4.2	7.1	4.7	9.8	5.5
4	4.1	4.3	3.9	4.2	4.2	7.1	8.4	9.8	3.2
5	4.4	4.3	3.9	5.0	4.2	7.1	10.2	9.8	8.3
6	4.1	4.3	3.9	4.3	4.2	7.1	4.0	9.8	5.8
7	4.5	4.3	3.9	5.1	4.2	7.1	9.3	9.8	6.2
8	5.6	15.0	3.0	3.9	27.0	2.5	5.2	15.9	6.0
9	6.0	16.0	3.7	3.9	20.2	6.0	5.2	16.9	7.8
10	7.1	20.2	4.1	3.9	42.4	5.5	5.2	20.2	11.3

RMSD and ligand RMSD values of the top 10 models for the full GroES-GroEL complex (in Å), compared to the reference structure; models were fitted (energy-minimized) using a 40 Å simulated density map and ranked by gradient matching using a 20 Å simulated density map. Then, the top 100 models were subjected to ring recombination; the resulting combinations were again ranked by gradient matching.

### Application to the full GroES-GroEL, experimental data

Finally, the fitting of the full GroES-GroEL complex was repeated, but now using a 23.5 Å experimental cryo-EM map (EM databank code 1046), adapting the experimental map to be compatible with our computational assembly protocol (see Materials and Methods).

**Figure 5:**
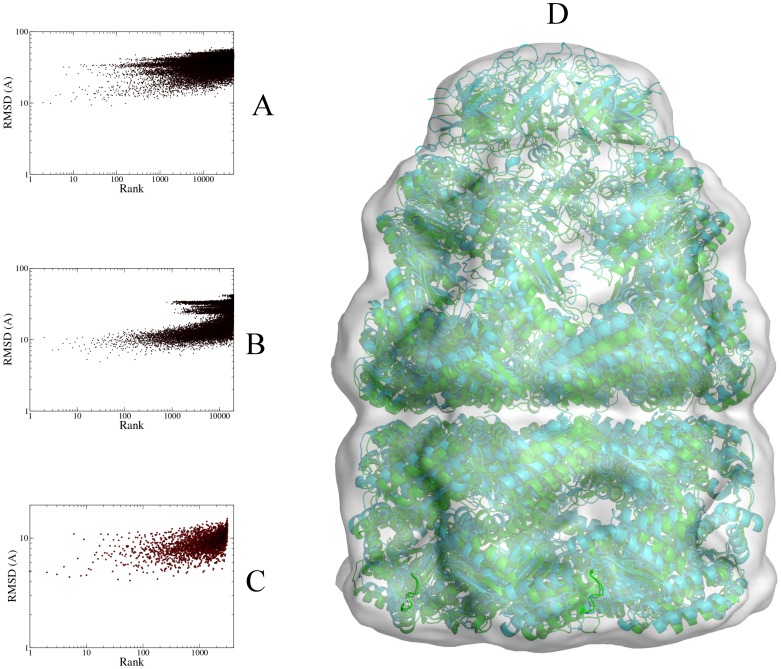
Assembly results for the full GroES-GroEL complex, using experimental data. A) RMSD values of generated models for the full GroES-GroEL complex, compared to the reference structure; models were assembled using a 23.5 Å experimental density map [35] that was downsampled to 44.8 Å; then, models were rescored with GVM using the full density map. B) RMSD values of recombined models for the full GroES-GroEL complex, compared to the reference structure; the top 100 models from A) were subjected to ring recombination; the resulting combinations were again rescored with GVM. C) RMSD values after two ring recombinations, repeating the procedure on the top 100 models from B). Note the different scaling on the logarithmic Y axis. D) Best-scoring refined structure (green), overlaid onto the reference structure (cyan) (RMSD: 4.6 Å); Top-scoring structures from C) were refined using the ATTRACT force field, and rescored with GVM. Image was generated with PyMol [63].

The initial sampling was somewhat poorer than for the simulated run, producing only 4 structures of RMSD better than 10 Å, the best structure being no better than 9.2 Å. We proceeded to score and re-rank all generated structures using GVM (figure 5a). Three out of four structures with RMSD<10 Å were ranked in the top 100 and one of them in the top 10 (table 6).

**Table 6:** Top 10 RMSD values for GroES-GroEL with experimental data, after the scoring stage.

	Overall RMSD			
			GroEL	GroEL
Rank	all	GroES	cis	trans
1	11.1	19.7	5.7	12.7
2	12.1	33.5	8.0	5.1
3	10.1	25.9	6.7	7.2
4	9.8	15.1	6.9	10.5
5	12.7	32.2	6.4	10.6
6	13.2	23.7	7.9	14.6
7	31.9	20.5	2.9	46.2
8	25.0	25.2	3.2	35.1
9	31.7	21.7	6.5	45.3
10	10.6	21.4	9.5	8.3

RMSD and ligand RMSD values of the top 10 models for the full GroES-GroEL complex (in Å), compared to the reference structure; models were fitted (energy-minimized) using a 23.5 Å experimental density map that was downsampled to 44.8 Å; then, the models were ranked by gradient matching using the full density map.

Breakdown of the RMSD values per component (table 6) shows that the cis ring was equally well assembled as in the simulated run (and GroES equally badly), but the trans ring much less so. Rings were again recombined, which improved the sampling (figure 5b), but much less than for the simulated run: in the top structures, both GroEL rings were now correctly assembled, but not GroES. Therefore, another round of ring recombination was carried out, taking again the top 100 structures and recombining them. This resulted in 3102 unique structures with an excellent sampling around the reference structure (figure 5c). Table 7 shows a breakdown of the top 10 in terms of RMSD. The top ranked structure has an overall RMSD of 5.0 Å, and all three rings are correctly assembled. The first six structures are all similar to the top ranked structure. All structures in the top 10 have the correct GroEL rings assembled, and only three of them have an incorrect GroES structure.

**Table 7:** Top 10 RMSD values for GroES-GroEL with experimental data, after ring recombination.

					ligand RMSD				
	Overall RMSD				intra-ring			inter-ring	
			GroEL	GroEL		GroEL	GroEL	cis-	cis-
rank	All	GroES	cis	trans	GroES	cis	trans	GroES	trans
1	5.0	7.5	4.3	5.1	2.7	1.9	0.9	5.8	3.3
2	5.0	7.5	4.1	5.1	2.7	5.9	0.9	7.7	5.1
3	4.9	7.5	3.8	5.1	2.7	2.7	0.9	6.7	2.5
4	4.7	7.5	3.2	5.1	2.7	6.9	0.9	10.2	10.6
5	5.5	7.5	5.3	5.1	2.7	3.8	0.9	3.5	9.5
6	4.4	7.5	3.8	4.2	2.7	2.7	8.2	6.7	6.6
7	10.9	32.7	4.3	5.1	28.4	1.9	0.9	31.2	3.3
8	6.5	15.9	4.3	5.1	19.6	1.9	0.9	16.2	3.3
9	4.5	7.5	4.1	4.2	2.7	5.9	8.2	7.7	7.3
10	9.7	29.2	4.3	5.1	18.8	1.9	0.9	27.1	3.3

RMSD and ligand RMSD values of the top 10 models for the full GroES-GroEL complex (in Å), compared to the reference structure; models were fitted (energy-minimized) using a 23.5 Å experimental density map that was downsampled to 44.8 Å; after that, the models were ranked by gradient matching using the full density map. Then, the top 100 models were subjected to ring recombination; the resulting combinations were again ranked by gradient matching. This recombination procedure was then repeated.

Finally, the top 1000 structures were refined with the full map and the ATTRACT force field. The top 10 structures are shown in table 8. As expected, overall RMSD did not change dramatically; as before, most, but not all, structures in the top 10 have the correct GroES ring, while both rings of GroEL are assembled correctly for all of them. In contrast, significant improvements were observed for the ligand RMSD, mostly within the GroEL cis ring and between the GroEL rings.

**Table 8:** Top 10 RMSD values for GroES-GroEL with experimental data, after refinement.

					ligand RMSD				
	Overall RMSD				intra-ring			inter-ring	
			GroEL	GroEL		GroEL	GroEL	cis-	cis-
rank	All	GroES	Cis	trans	GroES	cis	trans	GroES	trans
1	4.6	7.5	3.5	4.8	2.9	1.6	1.0	6.2	3.7
2	3.8	7.5	3.4	3.0	2.2	1.4	3.0	8.0	2.4
3	3.5	7.3	2.0	3.3	3.0	1.0	2.7	5.7	4.7
4	4.2	7.5	2.5	4.5	1.8	3.3	1.2	6.7	5.9
5	4.1	7.5	3.8	3.4	4.0	1.2	3.7	6.9	2.9
6	5.9	15.3	2.8	4.9	19.3	0.7	1.1	14.8	5.0
7	8.1	24.2	2.8	4.9	32.9	1.1	1.0	24.0	4.6
8	7.6	24.4	1.8	3.0	32.6	1.4	2.9	23.1	3.3
9	5.8	13.9	3.7	4.8	20.7	1.7	1.0	14.8	4.4
10	2.8	7.5	2.0	1.5	1.9	2.0	1.4	8.4	3.0

RMSD and ligand RMSD values of the top 10 models for the full GroES-GroEL complex (in Å), compared to the reference structure; models were fitted (energy-minimized) using a 23.5 Å experimental density map that was downsampled to 44.8 Å; after that, the models were ranked by gradient matching using the full density map. Then, the top 100 models were subjected to ring recombination; the resulting combinations were again ranked by gradient matching. This recombination procedure was then repeated. Finally, structures were refined using the ATTRACT force field, and again ranked by gradient matching.

The top-ranked structure is shown in figure 5d. Its overall RMSD is slightly improved to 4.6 Å, and a 2.8 Å structure was generated and ranked #10. The cis and trans rings are well-assembled, with RMSDs of around 1 Å, with intra-GroEL and cis-trans RMSDs around 3 Å. Note that for the cis ring, these RMSD values are no less accurate than for the first assembly run, even though in that run, only one ring was assembled, and simulated, noise-free data was used.

In contrast to the other interfaces (but in accordance with the run with simulated data), the small GroES-cis-GroEL interface could not be assembled with a (ligand) RMSD better than 5 Å, neither in the top structure nor in other top 10 structures. In fact, the improved assembly of the larger interfaces during refinement made the small GroES-cis-GroEL interface somewhat worse. In using low resolution cryo-EM data to assemble GroES-GroEL with the highest possible accuracy, modeling the GroES-cis-GroEL interface is clearly the limiting factor for our method.

In conclusion, we were able to generate a structural model for the GroES-GroEL complex with an overall accuracy of 4.6 Å RMSD, and an even better structure (2.8 Å) is present in the top 10. This is much better than the 14.7 Å structure that was generated by GMFit [29]. Comparing figure 4b and 5b, GVM scoring with experimental data works equally well for experimental and simulated maps. Although in both cases, initial sampling is poor, it is shown that sampling difficulties can be overcome by ring recombination, and that the resulting structures are selected by GVM, equally reliably with the experimental map as with simulated data.

### Additional experiments and controls on GroES-GroEL

In order to test the robustness of the method, two additional control experiments were performed on GroES-GroEL.

First, it was found that GVM scoring works even at very low resolution. Using only the experimental GroES-GroEL map filtered down to 44.8 Å resolution, a structure with 5.3 Å RMSD could be scored in the top 10 (see Supporting Analysis 4 in Supporting File S1). In general, non-reliance on structural details also indicates a (strong) resistance to structural noise.

Second, we performed a negative control by swapping the initial positioning of the cis and trans ring components in the starting structures. The resulting assembled models had a much poorer GVM score, showing that our method can distinguish between the correct and the wrong ring order (see Supporting Analysis 5 in Supporting File S1).

### Application to symmetric oligomers

ATTRACT-EM was also applied to four other complexes that have been used as test cases in existing methods for simultaneous multi-component cryo-EM fitting [29], [31] (table 9; more details about these complexes are provided in Supporting Analysis 1 in Supporting File S1). This revealed that the performance of ATTRACT-EM is overall similar to existing methods for these small complexes. ATTRACT-EM worked particularly well for the trimer (2NIC), and for the hexamer (2REC), even though in the latter case, no molecular refinement could be performed due to a lack of an atomic-resolution structure.

**Table 9:** Overview and comparison of all symmetric assembly results.

	Components	ATTRACT-EM	GMFit	IQP
1AON, cis ring	7 (C7)	2.5 (1.0)	-	8.6*
1AON, full	21 (C7)	4.6*	14.7*	-
1AFW	2 (C2)	6.8 (0.7)	1.0	0.9
2NIC	3 (C3)	0.6(0.3)	1.8	1.1
7CAT	4 (D2)	5.4	2.3	-
2REC	6 (C6)	2.9	2.3	1.0

Overview of all computational assembly results of ATTRACT-EM for symmetric assemblies, and comparisons to GMFit [29] and IQP [31]. All values are RMSDs in Å. Values in parentheses are ligand RMSDs. All results were obtained with 20 Å simulated maps, except the results marked with *, which were obtained with a 23.4 Å experimental map ([35], EMD code 1046). The “components” column, shows the number of components, and the symmetry of the complex within parentheses.

Molecular refinement could also not be performed for the tetramer (7CAT): the topology of the tetramer, containing long wrapping tails, led to more clashes in the initial models than the ATTRACT forcefield could deal with. The initial model had nevertheless a reasonable RMSD (5.4 Å), albeit worse than GMFit (2.3 Å). The performance for the dimer (1AFW) was disappointing; while the relative placement (0.7 Å ligand RMSD) was excellent, the absolute placement (6.8 Å) was inferior to other methods.

### Application to dimers

In principle, for ATTRACT-EM (or any fitting method), the fitting of small dimers is out of scope, since cryo-EM density maps cannot be obtained for complexes smaller than a few hundred kilodaltons. However, small dimers are the only available source of asymmetric complexes where the individual components have been crystallized in the free form (unbound structures). Therefore, to investigate the effect of symmetry and bound/unbound forms, ATTRACT-EM was applied to a number of small dimers. The 1AFW dimer was assembled with and without symmetry restraints. In addition, three non-symmetrical dimers from the protein-protein docking benchmark [70] were assembled, using their unbound crystal structures. For 1WQ1 (which has the highest conformational change of the three, belonging to the “medium” category of the docking benchmark), assembly using the bound forms was also tested for comparison.

Table 10 shows that the standard ATTRACT-EM protocol does not work very well for small dimers. For two dimers, 1AVX and 1AY7, the assembly failed completely. For the two other dimers, 1AFW and 1WQ1, partial success was achieved, with RMSDs of 5–6 Å in the top 10.

**Table 10:** Overview of the assembly results for dimmers.

	assembly stage		refinement stage		integrated protocol		location restraints	
	RMSD	l-RMSD	RMSD	l-RMSD	RMSD	l-RMSD	RMSD	l-RMSD
1AFW 1 ^,^ 3	6.3	5.7	6.8	0.7	0.3	0.7	4.3	6.4
top 10	5.9	4.5	6.8	0.7	0.3	0.6	4.0	3.0
all	5.9	3.3	6.8	0.7	0.3	0.2	4.0	1.7
1AFW 2 ^,^ 3	7.3	17.9	17.3	26.2	0.9	0.6	3.0	6.5
top 10	7.3	17.9	17.3	26.2	0.9	0.6	3.0	6.5
all	7.3	4.6	9.0	7.5	0.9	0.2	3.0	3.7
1AVX	38.8	28.4	-	-	16.4	25.0	3.9	6.3
top 10	22.7	25.8	-	-	10.6	13.5	3.9	5.0
all	13.2	2.9	-	-	10.1	1.8	3.9	3.2
1AY7	19.0	50.1	-	-	13.9	19.4	3.8	5.9
top 10	13.5	21.7	-	-	13.9	19.4	2.9	3.5
all	10.6	4.7	-	-	7.6	2.0	2.9	3.2
1WQ1	7.8	11.8	12.3	21.6	14.4	24.3	3.4	6.3
top 10	5.5	7.6	6.6	10.3	5.4	4.7	3.4	3.6
all	5.3	3.3	6.1	5.9	5.2	2.5	3.4	3.5
1WQ1^3^	12.3	20.3	4.5	6.8	3.8	0.8	6.9	11.6
top 10	5.3	7.0	4.5	6.8	3.8	0.8	3.1	4.6
all	4.9	4.0	4.5	1.2	3.8	0.8	3.1	1.7

Overview of all computational assembly results of ATTRACT-EM for symmetric assemblies. Indicated are the result for the top-scoring structure, the best result in the top 10 scoring structures, and the best result among all structures. All RMSDs are in Å. All complexes are unbound non-symmetric heterodimers unless indicated otherwise.

Note that true RMSDs were computed towards the bound complex: e.g. for 1WQ1, the theoretical best RMSD (ligand-RMSD) is 0 Å (0 Å) for the bound assembly but 1.7 Å (2.0 Å) for the unbound assembly, due to the conformational differences between bound and unbound forms.

1 = the complex is a symmetric homodimer; C2 symmetry restraints were used.

2 = the complex is a symmetric homodimer; C2 symmetry restraints were **not** used.

3 = the complex is in the bound form.

Nevertheless, sub-angstrom precision was achieved for 1AFW with an integrated protocol where the ATTRACT forcefield is included from the beginning. However, such an integrated protocol exploits the fact that the structures are in the bound form; this could also be seen for 1WQ1, with much better results for the bound form than for the unbound form. In contrast, the standard protocol (with separate assembly and refinement stages) seems to be robust for conformational change, with no differences in performance between bound and unbound forms.

For many molecular machines, the locations of one or more components are known. This knowledge can be obtained experimentally in a variety of ways, for example from a new cryo-EM experiment where a component has been genetically deleted or labeled with an antibody, or by cross-linking experiments between a component and other components of known location. To investigate the effect of such experimental knowledge, which is fuzzy and approximate, all dimers were tested again in the presence of very loose location restraints. This directed the monomers to specific locations in the map, corresponding to the true location with a large random displacement (average displacement of 4.8 Å). No further refinement of structures was performed.

The presence of these loose location restraints completely solved the sampling difficulties with small dimers: in all cases, in the initial assembly stage, structures of 3–4 Å RMSD were generated in the top 10, regardless which dimer and whether the bound form or the unbound form was assembled. In addition, for 1AFW, assembly without symmetry restraints performed no worse than with symmetry restraints, showing that location restraints are an effective substitute for symmetry in reducing the conformational space.

## Discussion

### The ATTRACT-EM protocol

Accurate prediction of the subunit arrangement in low resolution cryo-EM derived electron density maps is a challenging and still largely unsolved task. In the present study, a new approach, ATTRACT-EM, was presented that combines search strategies and techniques from the protein-protein docking field with cryo-EM derived data encoded as additional docking penalty score. ATTRACT-EM is based on the protein-protein docking program ATTRACT, and applies well-established concepts and techniques from the docking field to cryo-EM fitting: the separation of sampling (searching) and ranking (scoring), an atomic representation of molecular components, the rigid-body optimization of interactions using an intermolecular force field, and the use of experimental data to reduce the conformational space that is to be searched. It is customary in the protein-protein docking field to treat sampling (searching) and ranking (scoring) as two separate problems [66], and the same approach has been followed here. A good sampling method generates correct solutions among many others, and its score acts like a filter, eliminating highly improbable solutions. During the searching phase of the method (starting from millions of start arrangements) a smooth Gaussian overlap function between subunits and electron density is maximized to fit the subunits into the density. In addition, a search strategy was designed based on a systematic “recombination” of parts of predicted subunit arrangements, akin to the combinatorial approach followed by MultiFit [30].

During the ranking/rescoring phase of the protocol, a new gradient vector matching (GVM) algorithm was used to select most likely solutions. For the GroEL/GroES system, the GVM algorithm showed exceptional performance, consistently ranking the most accurate solutions at the top, for the cases that we tested. The GVM function was designed to be local, which means that in principle, forces to improve the matching can be computed, allowing the matching error to be minimized during the fitting process. However, we have not yet succeeded in integrating the gradient matching into the energy minimization process in a satisfactory way, and this will be the subject of future research.

### Noise tolerance of the GVM scoring

Comparable results were obtained for GroES-GroEL for the simulated and experimental maps. In general, the GVM scoring performed excellent at very low resolutions: 20 Å or worse, and to a large extent even at resolutions worse than 40 Å. While this is not particularly useful in the context of modern single-particle cryo-EM maps, which typically have a far better resolution, it does give a strong indication of noise tolerance. In a density fitting context, noise can be considered as discrepancy between the density map and the (simulated density of) the actual fitted protein coordinates, and this works in both directions. Clearly, at low resolution, the typical inaccuracies of a few angstroms in the structural coordinates have a much smaller impact on the overall electron density than at high resolution. Therefore, the fact that the GVM scoring method relies only on low-resolution features, indicates that it may be able to deal with *structural* noise as well, in the form of inaccurate protein coordinates. This is supported by our results on unbound dimers, which did not show any negative influence compared to the bound form. Still, this is a somewhat speculative interpretation which needs to be investigated more thoroughly in a future study.

### Performance of the method

The multi-stage protocol turned out to be a very effective strategy for the assembly of the GroEL/GroES test system. In combination with final molecular refinement, our method was able to generate much more accurate structures for GroES-GroEL (4.6 Å accuracy) than previous methods that used the same experimental cryo-EM density map. Similar good performance was also achieved for the 7-component cis ring of GroES-GroEL and other oligomeric complexes again including symmetry restraints. In contrast, considerable sampling problems were encountered for dimers. The smooth, 40 Å wide Gaussian overlap function used in sampling does an excellent job at the smoothening of energy barriers, preventing the computational assembly to get stuck in local energy minima. However, this comes at a loss of specificity that is manageable for large assemblies (the solutions merely have to be re-scored), but that prevents the sampling of correct solutions for dimers. The low resolution density alone (resolution similar to the size of the dimers) does not provide a sufficient driving force towards the native arrangement. We do not consider the poor performance on dimers to be a very large problem, since with current technology, cryo-EM density maps cannot be obtained for complexes smaller than a few hundred kilodaltons, and such complexes typically contain much more than 2 components. Still, two strategies to overcome the sampling problem for dimers were tested. One of them, additional restraints on the approximate location of the subunits, led to a large improvement in performance. In contrast, our results suggest that the other solution, the use of an atomic forcefield in the *initial* assembly, should be discouraged. For dimers, this led to unrealistically good results with bound starting structures that were not reproduced with the unbound forms. For larger assemblies, the use of an atomic force field in the initial assembly simply does not work because the energy landscape becomes too rugged and structures get stuck in local minima (results not shown).

In contrast, the multi-step assembly process used in ATTRACT-EM (initial assembly followed by molecular refinement) seems to be robust for (limited) conformational differences: no differences were found between bound and unbound forms, at least for the dimers that we tested. More research is needed to determine what conformational differences can be tolerated by the current protocol.

It needs to be emphasized that for all tested cases, the ATTRACT-EM approach at the current stage required additional data (besides the cryo-EM density), either in the form of symmetry or the approximate subunit locations in the assembly, to achieve results in close agreement with experiment. In the case of GroES-GroEL, these data can be derived from the density map itself, but this is not generally the case. Also, we must stress that all test cases in this study are computational exercises, where bound subunits and/or simulated density maps have been used. Improvements in the ATTRACT-EM method and protocol are clearly required before it can ultimately assemble a set of subunits simultaneously into an experimental density map without any additional restraints, in particular if these subunits are modeled from homologous components.

However, even at the current stage the approach can already be potentially very useful because in many cases additional experimental data on a multi-component complex is already available, in the form of cross linking data, distance restraints based on fluorescence energy transfer (FRET) or biochemical data on contacts between subunits in the assembly. For example, while the 26S proteasome consists of 33 components, the 14-component core particle has been wholly crystallized and can be easily placed into the map, and the arrangement and the approximate locations of nearly all other 19 components are known from cross-linking experiments [71]–[73]. Our results show that for dimers, location restraints are an excellent substitute for symmetry in reducing the conformational space; it is not known how well this scales to larger complexes.

### Optimization of parameters

More research will be needed to optimize protocol parameters, and to investigate their relation with the resolution and quality of the map and of the component structures. For example, for low resolution maps such as used here, a way must be found to deal with intermolecular spillover between the components (see Materials and Methods). While the global F parameter here is the most parsimonious solution, it is sub-optimal, since this spillover varies per protein region. However, ATTRACT-EM allows the expected overlap for each atom to be adjusted: in a real-world case, the biologist can use e.g. knowledge about interacting protein regions to model intermolecular spillover realistically.

### Atomic representation and flexibility

Unlike previous methods, ATTRACT-EM does not rely on any kind of feature detection or simplified representation of the components that are to be fitted; in contrast, atoms are represented explicitly in our method, albeit at a coarse-grained level. Therefore, our assembly protocol, in particular the molecular refinement stage (but not the GVM scoring), assumes some degree of accuracy in the atomic details, more so than other methods that use a less detailed representation. This can be problematic in case of inaccurate protein structures that undergo conformational change or that are modeled from inaccurate homologous structures. This problem is well known in the protein docking field [60], and is an ongoing area of research. In its present form, ATTRACT-EM has not been tested (and is unlikely to work well) with such structures. However, an explicit atomic representation makes our method also well suited to flexible fitting, which attempts to overcome and correct these inaccuracies. In fact, the ATTRACT docking program already natively supports flexible docking [51], [52] in a way similar to several flexible fitting methods [7], [8], namely in the form of normal modes. In ATTRACT, normal modes can be sampled efficiently as additional degrees of freedom in the energy minimization protocol. In addition, it is possible to represent parts of the subunits as multiple conformational copies and the docking score automatically selects the best-fitting copy during the search. While we have not used this option in this study, it is perfectly possible to combine normal modes and cryo-EM data in ATTRACT into a flexible fitting protocol, and this will be explored in a future study.

By building upon these concepts, ATTRACT-EM covers a middle ground between rigid fitting methods and flexible fitting methods for cryo-EM data, in terms of speed, accuracy and level of detail. On the one hand, the ATTRACT force field provides a physics-based, near atomic-resolution representation and energy function, in contrast to a simple electron density cross-correlation. ATTRACT-EM is not a fitting program: while the computational assembly process is guided by the cryo-EM map, its main purpose is to restrict the conformational space to regions that are in overall agreement with the cryo-EM data. Therefore, ATTRACT-EM is meant to supplement existing fitting programs, adding near atomic resolution, typically provided by flexible fitting programs at the refinement stage, already to the initial assembly stage.

While in the present application to GroEL/GroES the final structures were already in close agreement with the native complex structure, even without a fully flexible refinement stage the approach can potentially be used to generate a limited set of sterically favorable start conformations for a final fully flexible molecular dynamics fitting procedure. Thus, ATTRACT-EM is also suitable to be used as one part in a tool chain, consisting of programs for modeling, rigid fitting, flexible fitting, and/or molecular dynamics.

### Computational requirements and availability

Unlike existing methods for simultaneous computational assembly of molecular machines into cryo-EM map, ATTRACT-EM uses an atomic representation throughout the protocol and physics-based intermolecular interaction in the later stages. However, this increased level of detail and realism comes at the cost of computational efficiency: a typical ATTRACT-EM run takes a few dozen to a few hundred CPU hours to complete. ATTRACT and ATTRACT-EM are implemented in C++ and Fortran 95, with a number of Python utilities, and runs on any platform where these languages are available. The method will be available under the GPL license as part of ATTRACT 2.0, which is currently under development. In the meantime, the source code is available from the authors upon request.

## Supporting Information

Supporting Information File S1 **Contains Supporting Analysis 1–6, Supporting Tables S1–S4 and Supporting Figures S1–S4.**
(DOC)

Movie S1 **ATTRACT-EM pre-assembly stage.** The structure shown is the best-scoring model of the cis ring of GroES-GroEL, using simulated electron density.
(AVI)

Movie S2 **ATTRACT-EM assembly stage.** The structure shown is the best-scoring model of the cis ring of GroES-GroEL, using simulated electron density.
(AVI)

Movie S3 **ATTRACT-EM molecular refinement stage.** The structure shown is the best-scoring model of the cis ring of GroES-GroEL, using simulated electron density.
(AVI)
