# Supplementary material for: ATTRACT-EM: A New Method for the Computational Assembly of Large Molecular Machines Using Cryo-EM Maps
Source: PLoS One. 2012 Dec 14;7(12):e49733. doi: 10.1371/journal.pone.0049733 (PMC3522670; doi:10.1371/journal.pone.0049733)
Supplement: Supporting Information File S1 — Contains Supporting Analysis 1–6, Supporting Tables S1–S4 and Supporting Figures S1–S4. (DOC) [file pone.0049733.s001.doc]

**Supporting Information File S1**

**Supporting analysis 1**

*Application of the method to other complexes*

As a control, ATTRACT-EM was applied to four other complexes, previously assembled by the simultaneous-assembly methods GMFit [34] and IQP [36].

As usual, initial assembly (500 000 structures) with a 40 Å simulated density map was followed by GVM re-scoring with a 20 Å map. The top 1000 structures were then refined with the standard refinement protocol, including the ATTRACT force field (except for 7CAT and 2REC, see below).

The results are summarized in table 9 of the main text, and are discussed in detail below:

*1AFW (dimer, C2 symmetry)*

Despite the fact that 1AFW consists only of two components, assembly using completely random starting structures failed (RMSD values no better than 8 Å); instead, random starting structures were generated with perfect C2 symmetry around the actual symmetry axis. Even then, symmetry became violated during the assembly stage. This shows that our symmetry distance restraints are not strong (or numerous) enough for the C2 case, since the number of equivalent distances is much smaller than for higher C symmetries. This symmetry violation was prevented by repeating the C2 symmetry specification ("-symmetry 1 2" option in ATTRACT) two times, effectively tripling the strength of the symmetry restraints.

After the initial assembly and GVM rescoring, the top-ranked structure had an overall RMSD of 6.32 Å; the RMSDs in the top 10 ranged from 5.85 to 29.87 Å, with nine out of ten structures under 8 Å. In terms of ligand RMSD, the top-ranked structure was at 5.74 Å, and the top 10 ranged from 4.55 Å to 65.08 Å.

Refinement of the structures resulted in dramatic improvement in the relative placement of the components, as measured by their ligand RMSDs: the top 21 structures with the best ATTRACT score all had a ligand RMSD of better than 1 Å (0.67-0.84 Å). Unfortunately, the refinement did not improve the absolute placement of the components. Ranked by GVM energy, the top-ranked refined structure has a sub-angstrom ligand RMSD (0.704 Å) but an overall RMSD of 6.80 Å, which is also the best overall RMSD of all 1000 refined structures.

In terms of relative placement, the achieved sub-angstrom accuracy is comparable with the RMSDs of GMFit (1.03 Å overall RMSD) and IQP (0.9 Å overall RMSD). However, unlike for GMFit, C2 symmetry is needed to obtain this result with ATTRACT-EM. More importantly, in terms of absolute placement, the 6.80 Å of ATTRACT-EM is comparable to the results for GroES-GroEL, but much worse than the accuracy of GMFit and IQP for 1AFW.

*1NIC (trimer, C3 symmetry)*

In the case of 1NIC, it was not necessary to pre-generate random starting structures with the correct symmetry: starting from completely random orientations, the imposed C3 distance restraints led automatically to structures with the correct symmetry and good RMSD during the assembly stage. Out of all 500 000 structures, 11 structures had an RMSD under 6 Å and 220 structures under 10 Å. As in all other test cases, GVM scoring did an excellent job in selecting the correct structures: the top structure had an RMSD of 4.55 Å (ligand RMSD 6.43 Å). The top 10 contained a single wrong structure (RMSD 32.55 Å), with the remaining 9 structures between 4.55 and 7.57 Å.

Refinement for 1NIC worked very well in improving both the relative and absolute placement of the components. Seven structures with sub-angstrom ligand RMSD were generated and ranked at the top in ATTRACT score. After ranking the refined structures by GVM energy, a structure with 0.55 Å overall RMSD and 0.29 Å ligand RMSD was ranked first. This was by far the most accurate structure among the refined structures; in fact, the 1000 refined structures contained only 4 structures under 5 Å RMSD, and these structures were ranked 1-4.

For 1NIC, the sub-angstrom (0.55 Å) accuracy model generated by ATTRACT-EM is slightly more accurate than the models generated by GMFit (1.77 Å) and IQP (1.1 Å)

*7CAT (tetramer, D2 symmetry)*

D2 symmetry was implemented into ATTRACT ("symmetry -4 1 2 3 4" option).

Assembly using completely random starting structures failed, and random starting structures were generated with perfect D2 symmetry around the actual symmetry axes.

7CAT is a particularly difficult case for an atomic-resolution method such as ATTRACT. Each monomer has not only an extensive interface with two neighboring subunits, but also a long N-terminal tail (residue 3-68) that inserts itself into the interface between neighbor 1 and the fourth monomer, and a long loop region (residue 380-416) that wraps itself around neighbor 2. Despite this, ATTRACT-EM was able to generate some very reasonable structures: out of the 500 000 assembled models, 5 structures were under 6 Å and 33 were under 10 Å. GVM scoring reliably ranked these at the top: the top-ranked structure had an RMSD of 5.46 Å; among the top 10, seven structures were of good quality whereas the remaining three ones were wrong (top 10 RMSD range: 4.51 - 48.69 Å). However, all structures of low RMSD had their loop and tail regions penetrating deeply into the other monomers, leading to extremely high ATTRACT force field energies for the refinement stage. Therefore, only the first stage of the refinement (re-minimization with the 20 Å map) was carried out, with only minimal effect: the RMSD improved from 5.46 Å to 5.42 Å. While this comparable to our results for GroES-GroEL, it is worse than the 2.27 Å model of 7CAT produced by GMFit.

Another assembly run was carried out with the N-terminal tail and the loop region removed from the atomic structure (not changing the simulated electron density maps), but this did not lead to better results (results not shown).

*2REC (hexamer, C6 symmetry)*

2REC has not been solved at atomic resolution, but was deposited as a C-alpha trace derived from cryo-EM data [81]. Since then, the same structure has been crystallized as a helical filament ([82,83], PDB code 1XMV). To obtain an atomic-resolution reference structure, a filament monomer was superimposed six times onto the C-alpha trace. This reference structure was also used to generate simulated density maps. However, the packing between the monomers was of very poor quality: several clashing side chains had to be shaved, and even then the reference structure was unstable: subjecting it to the ATTRACT-EM refinement protocol led to a shift of 4.6 Å RMSD. Therefore, it was decided to truncate the refinement protocol for generated models, and only re-minimize them with the 20 Å cryo-EM density map, not using the ATTRACT force field.

Assembly using completely random starting structures failed, and random starting structures were arranged in a hexagon ring around the actual symmetry axis, followed by a rotational minimization (with symmetry restraints only) to impose C6 symmetry.

Initial assembly followed by GVM re-scoring yielded a top-ranking structure of 3.10 Å, and the complete top 10 was better than 6 Å (2.67 Å - 5.21 Å). Re-minimization with the 20 Å led to small improvements, with the top-ranking model now at 2.85 Å. This accuracy is comparable to GMFit (2.3 Å for 2REC), and slightly less accurate than IQP (1.0 Å for 2REC).

**Supporting analysis 2**

*Refinement of initial models using a 20 Å map, without a GVM scoring stage*

The assembly stage of the GroEL cis ring, using simulated data, resulted in initial assembled models, several of which were better than 10 Å (see main text, figure 2a).

A refinement of these models was carried out using a 20 Å data: the fitting energy to the 20 Å map was added as an additional term to the total energy, and the structures were re-minimized. Figure S2 and table S1 show the results for the refinement of the best 20 000 initial models. Overall RMSD and ranking showed an improvement: 87 structures had an RMSD of better than 10 Å, the lowest RMSDs being 4.0 Å (rank 36) and 4.3 Å (rank 19). Still, although the top 10 contains several accurate structures, the results were not perfect: the highest-scoring solution has the correct interface (4.9 Å ligand RMSD), but the structure is rotated in the ring plane, leading to an overall wrong placement (18.2 Å overall RMSD). Several other structures in the top 10 are wrong altogether, corresponding to flipped or inverted solutions.

**Supporting analysis 3**

*Correlation analysis of predicted and experimental gradient vectors*

The Gradient Vector Matching (GVM) scoring algorithm compares for each voxel the gradient vector *V* derived from the electron density map (*V_exp_*) with the same *V* derived from the atom occupancies of the model (*V_model_*). To assess the discriminatory power of GVM for scoring, *V_exp_* and *V_model_* were compared in figure S3, for a number of complexes.

Figures S3 (left) shows *V_exp_* versus *V_model_* (all x, y and z components pooled) for three cis-ring GroEL structures: the reference structure, the best initial model (4.7 Å), and a completely wrong model (RMSD 55.4 Å, rank #2 according to the original total energy). The models come from the initially fitted structures, but the gradient vectors were calculated using the 20 Å map. The best model has a correlation coefficient almost as high as the reference structure, but the totally wrong model has almost no correlation at all between *V_exp_* and *V_model_*. Clearly, for the cis ring, high correlation is a strong indicator of a correctly assembled cis ring.

The analysis was repeated for the full GroES-GroEL complex with simulated data, again comparing three structures (figure S3 middle): the reference structure, the best initial model (8.4 Å), and a completely wrong model (RMSD 37 Å; rank #1 according to the original total energy). Although the best model is more than 8 Å away from the reference, the correlation coefficient is still better than 0.6, while there is again basically no correlation for the wrong model. These plots indicate that GVM works equally well for the full GroES-GroEL complex as for the cis ring.

Finally, correlations were analyzed for the full GroES-GroEL complex with experimental data (figure S3 right). Note that the reference structure is the same as in S3 middle, but the correlation drops from 0.89 to 0.75 due to experimental noise. However, the correlation for the best generated model (as far away as 9.2 Å!) is not diminished at all. This indicates that GVM is exceptionally tolerant to noise, both experimental noise in the density map and “noise” caused by the fact that the best model is still far away from the reference. Clearly, very close GVM agreement (in this case, a correlation better than 0.75) should not be aimed for, but the fact that wrong models consistently have a very low correlation shows the predictive power of the method.

**Supporting analysis 4**

*GVM scoring with 44 Å experimental map*

Throughout the study, we have used very low resolution maps (40 or 44.8 Å resolution) for the initial assembly stage. We wondered to what extent this very low resolution is sufficient also for the GVM scoring. For this, we re-scored the initial assembled structures (full GroES-GroEL complex, experimental map) with the GVM scoring using the same 44.8 Å map that was used to assemble the structure.

Initial GVM scoring is surprisingly good (figure S4, top; table S3); structures around 10 Å are ranked near the top, and the cis ring is assembled with excellent accuracy. There are some structures were the trans ring is also good (RMSD 10.7 Å, rank #2; also #5 , #9), although most of the top 10 structures have the trans ring upside down (RMSD 33.7 Å, rank #1; also #3, #4, #6, #7, #8).

In addition, ring recombination helped a lot to improve the sampling, without affecting the scoring too much (Figure S4, bottom; table S4). Rank #1 and #2 still have the wrong trans ring, but there is a 9.1 Å structure at rank #4, a 7.1 Å structure at rank #7, and even a 5.3 Å structure at rank #9. Note that this structure is comparable to the best structure using the full experimental density map!

In the case of 44.8 Å data, a second round of ring combination shows to be detrimental for scoring; the wrong structures that were #1 and #2 after the first round of recombination come to dominate the most favored structures. Although sampling is improved in the sense that structures under 5 Å are now generated, they all rank relatively poorly: the best structure at RMSD 4.5 Å comes at rank #481 (out of 3242). However, even this second, detrimental round of recombination still has the cis ring in the correct conformation.

**Supporting analysis 5**

*Negative control with swapped ring order*

When generating starting structures, the correct ring order GroES – *cis* – *trans* was assumed. This is obvious for the small GroES ring, which can be clearly identified as the small cap region of the experimental GroES-GroEL map. However, the *cis* and *trans* rings are similar in size, and from the cryo-EM map it is not obvious which one is in the middle and which is at the bottom. Therefore, as a negative control, another run was performed with the experimental map, but with the *cis* and *trans* components swapped in the starting conformations, to see if the fitting and the GVM scoring could discriminate between the two. All steps and calculations were completely identical between the two runs: the only difference is that for the alternative run, taking the starting conformations of the normal run, the *cis* and *trans* rings were swapped prior to assembly.

The top 100 structures in the normal run had a GVM energy of 1711 +/- 144 (standard deviation). In comparison, the top 100 in the alternative run had an energy of 2337 +/- 112. No structures from the alternative run would have made it into the top 100 of the correct run. Therefore, the GVM score clearly favors the correct arrangement.

**Supporting analysis 6**

*Standard atom density analysis*

The ATTRACT reduced-atom force field keeps all backbone heavy atoms (although some of them are dummy atoms that do not have any interaction), while the side chain heavy atoms are replaced by one or two beads (pseudo-atoms). In ATTRACT-EM, all (pseudo-)atoms are treated equally in the fitting. This means that the average mass of a pseudo-atom is lower for glycin, where each heavy atom is represented explicitly, than for e.g. tryptophan, where 14 atoms are represented by 6 beads. Nevertheless, the average mass of all pseudo-atoms in the protein is quite constant between proteins, e.g. 20.61 Da for trypsin and 20.75 Da for GroEL chain A.

Tsai et al. (1999) analyzed the packing density of various atom types. On the whole-protein level, they found an average density of 0.173 ml/g for 12 proteins, with a few percent variability. This corresponds to 0.287 A^3^/Da, or 287 Da per 10x10x10 A voxel. Using the average pseudo-atom masses computed above, this leads to an average atom pressure of 14 (13.8 - 13.9) pseudo-atoms / voxel. However, some fluctuations can be observed due to local differences in amino acid composition.

**Supporting tables**

| Rank | Overall RMSD | ligand RMSD |
| --- | --- | --- |
| 1 | 18.2 | 4.9 |
| 2 | 55.3 | 69.5 |
| 3 | 6.1 | 3.2 |
| 4 | 7.5 | 10.7 |
| 5 | 30.3 | 57.7 |
| 6 | 57.5 | 69.6 |
| 7 | 13.3 | 15.3 |
| 8 | 14.5 | 6.6 |
| 9 | 6.2 | 9.1 |
| 10 | 18.4 | 14.0 |

**Table S1**

RMSD and ligand RMSD values of the top 10 models for the GroEL cis ring, compared to the reference structure; models were fitted (energy-minimized) using a 40 Å simulated density map, then refined with a 20 Å simulated density map, and ranked by the same energy.

**Table S2**

|  | **Overall RMSD** | | | |
| --- | --- | --- | --- | --- |
| Rank | All | GroES | GroEL | GroEL |
|  |  |  | cis | trans |
| 1 | 37.0 | 27.5 | 39.5 | 36.0 |
| 2 | 17.7 | 18.1 | 18.0 | 17.2 |
| 3 | 41.7 | 25.1 | 38.1 | 47.3 |
| 4 | 37.2 | 29.1 | 35.9 | 39.8 |
| 5 | 17.5 | 24.3 | 12.8 | 19.8 |
| 6 | 16.1 | 25.1 | 11.7 | 17.6 |
| 7 | 14.2 | 34.0 | 5.6 | 13.3 |
| 8 | 26.2 | 23.6 | 16.2 | 33.6 |
| 9 | 33.4 | 23.9 | 27.0 | 33.0 |
| 10 | 34.0 | 13.9 | 35.8 | 34.9 |

RMSD values of the top 10 models for the full GroES-GroEL complex, compared to the reference structure; models were fitted (energy-minimized) using a 40 Å simulated density map and ranked by the same energy.

**Table S3**

|  | **Overall RMSD** | | | |
| --- | --- | --- | --- | --- |
| rank | All | GroES | GroEL | GroEL |
|  |  |  | cis | trans |
| 1 | 33.7 | 27.3 | 3.9 | 48.1 |
| 2 | 10.7 | 21.7 | 5.5 | 11.3 |
| 3 | 31.9 | 20.5 | 2.9 | 46.2 |
| 4 | 33.3 | 25.5 | 7.1 | 47.3 |
| 5 | 11.1 | 19.7 | 5.7 | 12.7 |
| 6 | 32.1 | 23.0 | 7.9 | 45.6 |
| 7 | 33.3 | 16.6 | 9.9 | 47.5 |
| 8 | 33.0 | 27.7 | 2.2 | 47.2 |
| 9 | 13.0 | 20.5 | 11.8 | 12.0 |
| 10 | 24.3 | 27.5 | 4.6 | 33.5 |

RMSD and ligand RMSD values of the top 10 models for the full GroES-GroEL complex, compared to the reference structure; models were fitted (energy-minimized) using a 23.5 Å experimental density map that was downsampled to 44.8 Å; then, the models were ranked by gradient matching using the 44.8 Å density map.

**Table S4**

|  | **Overall RMSD** | | | |
| --- | --- | --- | --- | --- |
| rank | All | GroES | GroEL | GroEL |
|  |  |  | cis | Trans |
| 1 | 31.9 | 11.2 | 3.3 | 46.6 |
| 2 | 32.8 | 11.2 | 3.3 | 48.1 |
| 3 | 11.9 | 27.5 | 4.6 | 12.0 |
| 4 | 9.1 | 11.2 | 3.3 | 12.0 |
| 5 | 11.6 | 27.5 | 4.6 | 11.3 |
| 6 | 32.3 | 11.2 | 3.3 | 47.3 |
| 7 | 7.1 | 11.2 | 3.3 | 8.6 |
| 8 | 32.7 | 27.7 | 2.2 | 46.6 |
| 9 | 5.3 | 11.2 | 3.3 | 5.1 |
| 10 | 8.7 | 11.2 | 3.3 | 11.3 |

RMSD and ligand RMSD values of the top 10 models for the full GroES-GroEL complex, compared to the reference structure; models were fitted (energy-minimized) using a 23.5 Å experimental density map that was downsampled to 44.8 Å; then, the models were ranked by gradient matching using the 44.8 Å density map. Finally, the top 100 models were subjected to ring recombination; the resulting combinations were again ranked by gradient matching.

**Supporting figures**

**
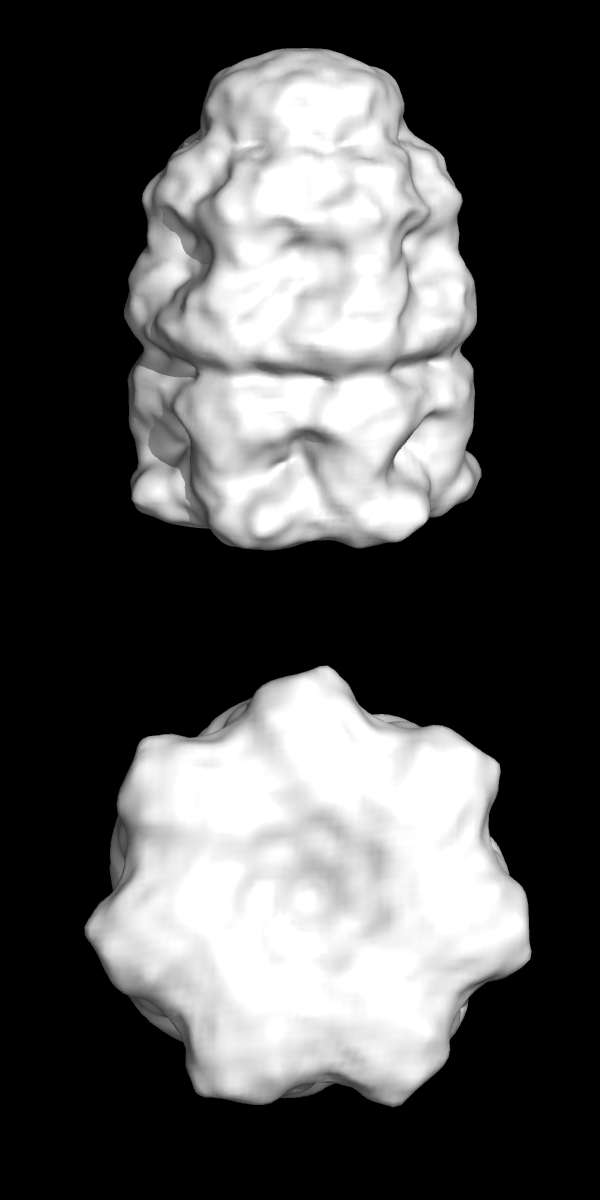
**

**Figure S1. The experimental cryo-EM density map at 23.5 Å resolution.**

Ranson et al. ([35]; EMD code 1046) . Image was generated with PyMol [63].

**
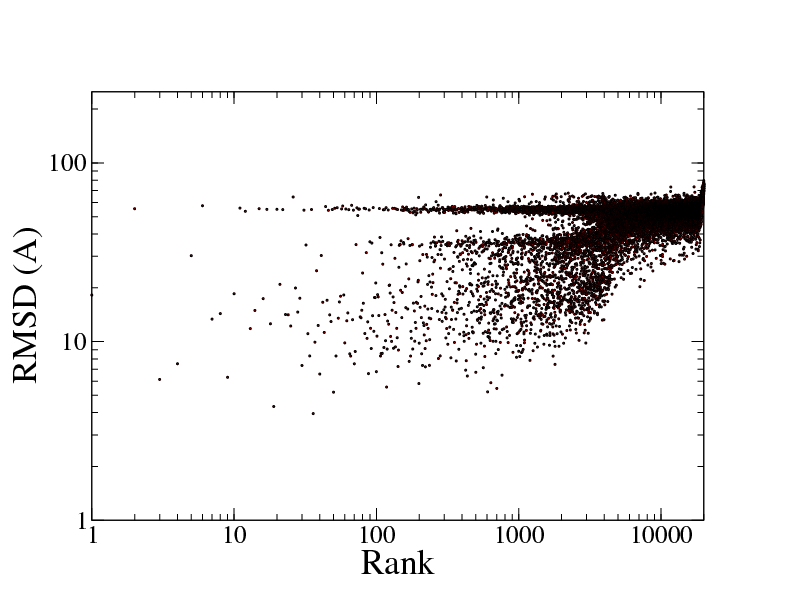
**

**Figure S2. Initial refinement results for the GroEL complex cis ring, using simulated data.**

RMSD values of the all generated models for the GroEL cis ring, compared to the reference structure; models were fitted (energy-minimized) using a 40 Å simulated density map, then refined with a 20 Å simulated density map, and ranked by the same energy.


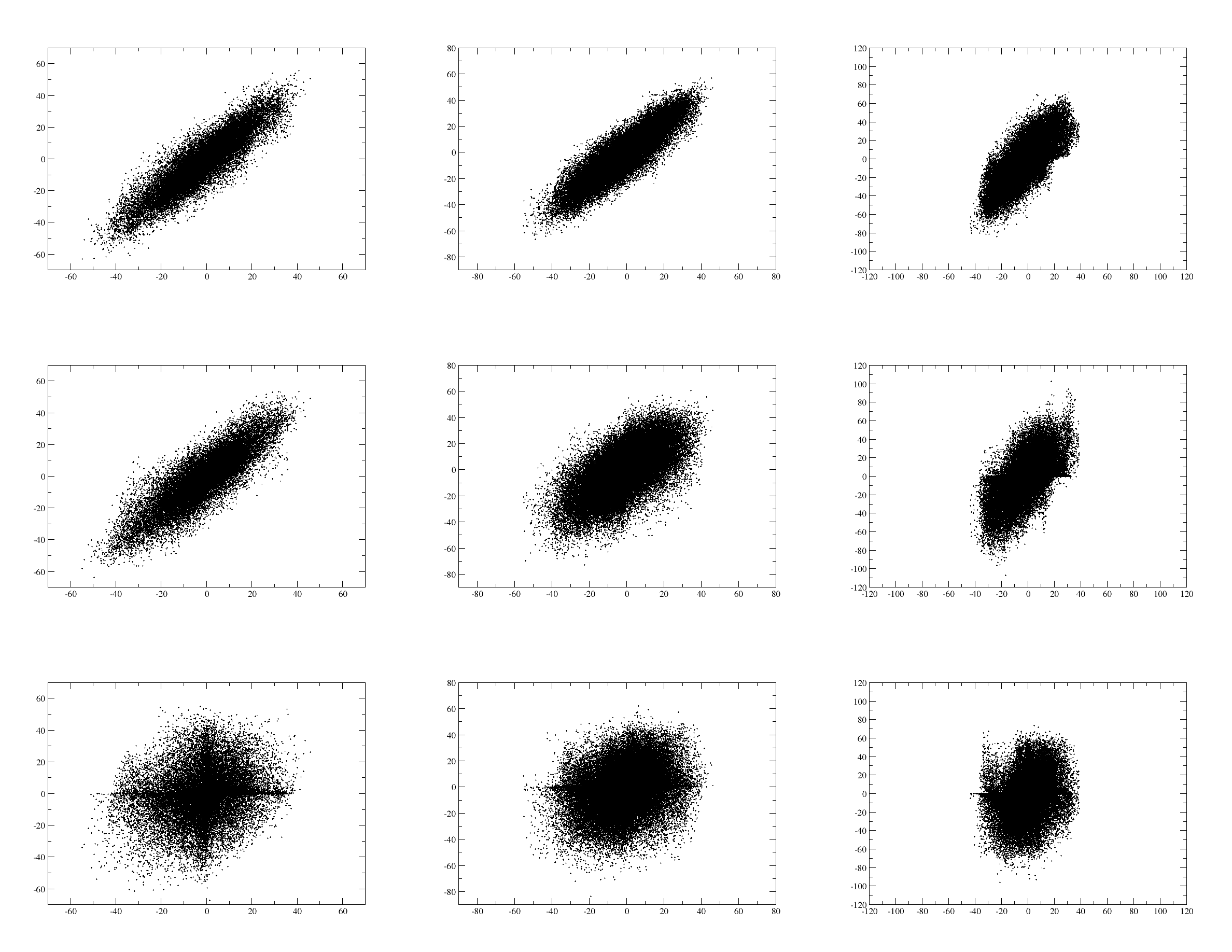


**Figure S3. The predictive value of gradient vectors.**

Correlation plots between experimental (simulated) gradient vectors and model gradient vectors. Values for the x, y and z components are pooled. Gradient vectors on the Y axis are derived from the electron density maps. Gradient vectors on the Y axis are derived from the voxel atom densities for the various models.

Left column: GroEL cis ring using simulated data. The assessed models are the reference structure (top), the best generated structure (RMSD 4.7 Å, middle), and a completely wrong structure (55.1 Å, bottom; this structure was ranked #2 in the original total energy). Correlation coefficients are 0.899, 0.859, and 0.198 respectively.

Middle column: full GroES-GroEL complex using simulated data. The assessed models are the reference structure (top), the best generated structure (RMSD 8.4 Å, middle), and a completely wrong structure (37.0 Å, bottom; this structure was ranked #1 in the original total energy). Correlation coefficients are are 0.895, 0.653, and 0.265 respectively.

Right column: full GroES-GroEL complex using an experimental density map [35]. The assessed models are the reference structure (top), the best generated structure (RMSD 9.2 Å, middle), and a completely wrong structure (47.2 Å, bottom; this structure was ranked #1 in the original total energy). Correlation coefficients are 0.751, 0.604, and 0.224 respectively.


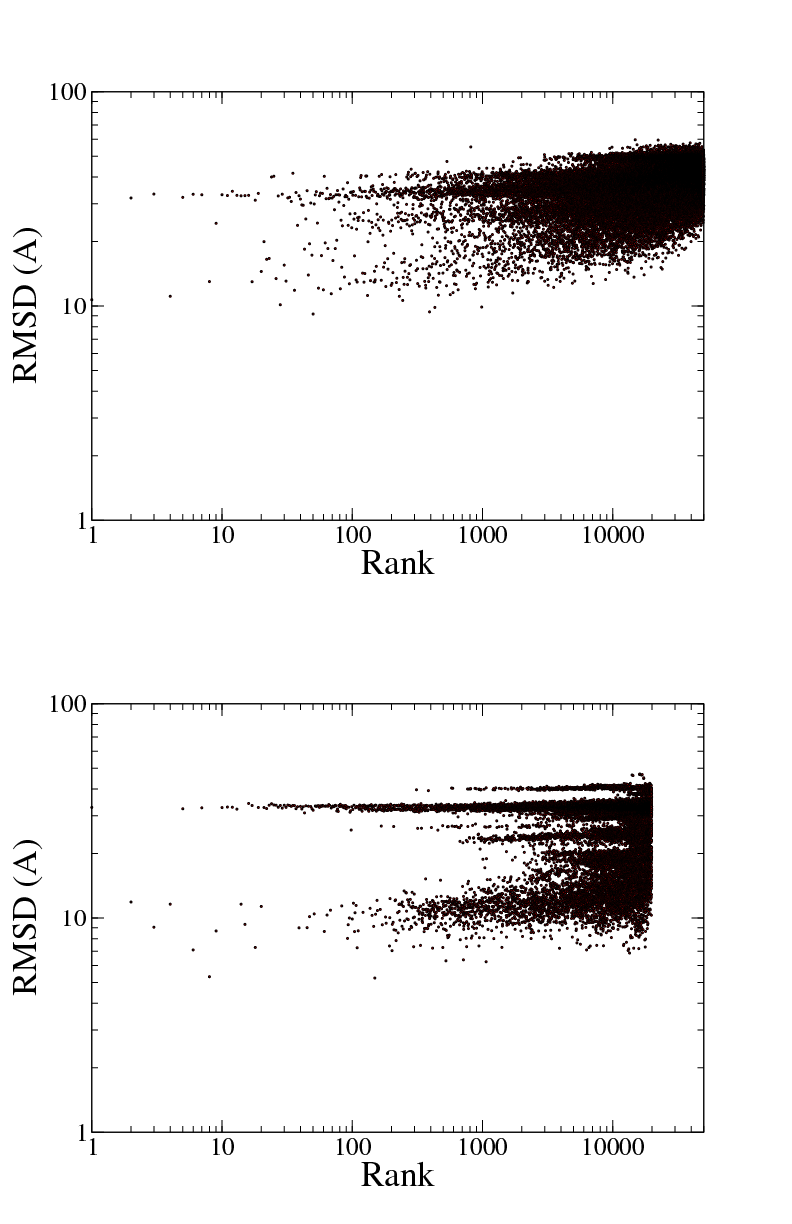


**Figure S4. Assembly results for the full GroES-GroEL complex, using downsampled experimental data.**

RMSD values of all generated models for the full GroES-GroEL complex, compared to the reference structure; models were fitted (energy-minimized) using a 23.5 Å experimental density map that was downsampled to 44.8 Å; then, the models were ranked by gradient matching using the same 44.8 Å density map (top). Then, the top 100 models were subjected to ring recombination; the resulting combinations were again ranked by gradient matching (bottom).
